# Cutting-Edge Progress in Stimuli-Responsive Bioadhesives: From Synthesis to Clinical Applications

**DOI:** 10.3390/polym14091709

**Published:** 2022-04-22

**Authors:** Elham Khadem, Mahshid Kharaziha, Hamid Reza Bakhsheshi-Rad, Oisik Das, Filippo Berto

**Affiliations:** 1 Department of Materials Engineering, Isfahan University of Technology, Isfahan 84156-83111, Iran; khadem_77@yahoo.com; 2 Advanced Materials Research Center, Department of Materials Engineering, Najafabad Branch, Islamic Azad University, Najafabad, Iran; rezabakhsheshi@pmt.iaun.ac.ir; 3 Structural and Fire Engineering Division, Department of Civil, Environmental and Natural Resources Engineering, Luleå University of Technology, 97187 Luleå, Sweden; oisik.das@ltu.se; 4 Department of Mechanical and Industrial Engineering, Norwegian University of Science and Technology, 7491 Trondheim, Norway

**Keywords:** bioadhesive, stimuli-responsive materials, wound healing, drug delivery

## Abstract

With the advent of “intelligent” materials, the design of smart bioadhesives responding to chemical, physical, or biological stimuli has been widely developed in biomedical applications to minimize the risk of wounds reopening, chronic pain, and inflammation. Intelligent bioadhesives are free-flowing liquid solutions passing through a phase shift in the physiological environment due to stimuli such as light, temperature, pH, and electric field. They possess great merits, such as ease to access and the ability to sustained release as well as the spatial transfer of a biomolecule with reduced side effects. Tissue engineering, wound healing, drug delivery, regenerative biomedicine, cancer therapy, and other fields have benefited from smart bioadhesives. Recently, many disciplinary attempts have been performed to promote the functionality of smart bioadhesives and discover innovative compositions. However, according to our knowledge, the development of multifunctional bioadhesives for various biomedical applications has not been adequately explored. This review aims to summarize the most recent cutting-edge strategies (years 2015–2021) developed for stimuli-sensitive bioadhesives responding to external stimuli. We first focus on five primary categories of stimuli-responsive bioadhesive systems (pH, thermal, light, electric field, and biomolecules), their properties, and limitations. Following the introduction of principal criteria for smart bioadhesives, their performances are discussed, and certain smart polymeric materials employed in their creation in 2015 are studied. Finally, advantages, disadvantages, and future directions regarding smart bioadhesives for biomedical applications are surveyed.

## 1. Introduction

An increase in the cost of healthcare and the age of the population has resulted in a rising request for bioadhesives and drug delivery systems [1]. The term “bioadhesion” was introduced for the first time in the 1970s. Bioadhesion is defined as the phenomenon in which two materials, one of which should be biological, are attached for a long time by interfacial tailoring [2]. Due to biodegradability, biocompatibility, and large molecular weight, bioadhesives can be applied in many hard-and soft-tissue applications, drug delivery, reinforcing fragile tissues in therapy, and helping with hemostasis [3]. Despite the potential benefits of bioadhesives, the functionality of commercially available bioadhesives is limited [4]. For example, existing adhesives are designedto support the injured tissues mechanically. To further explore the potential of bioadhesives in medicine, scientists have developed multifunctional bioadhesives with antimicrobial ability to limit microbial adherence and growth [5,6]. Additionally, some bioadhesives with self-healing capabilities can prolong the adhesive’s available time [7]. However, nearly all common materials are in a static condition when utilized as bioadhesives; “smart”, “intelligent”, or “stimuli-responsive” bioadhesives have been introduced [8]. Smart bioadhesives are generally defined as materials that can sense and react to different stimuli, including photoradiation (UV, visible light), temperature, pH, specific ions, solvents, electric and magnetic fields, redox conditions, mechanical stress, and biomolecules. Smart bioadhesives are of great interest for treatment systems where they can be used to control the release of drugs, close wounds, and fix devices on/in the body through non-invasive methods without damage until they have reached their desired aim. They are not only inexpensive, but they are also easy to control. Therefore, smart bioadhesives with this potential can change their performance and adhesive behavior in response to changing physiological conditions and promote treatment processes [9].

**Figure 1:**
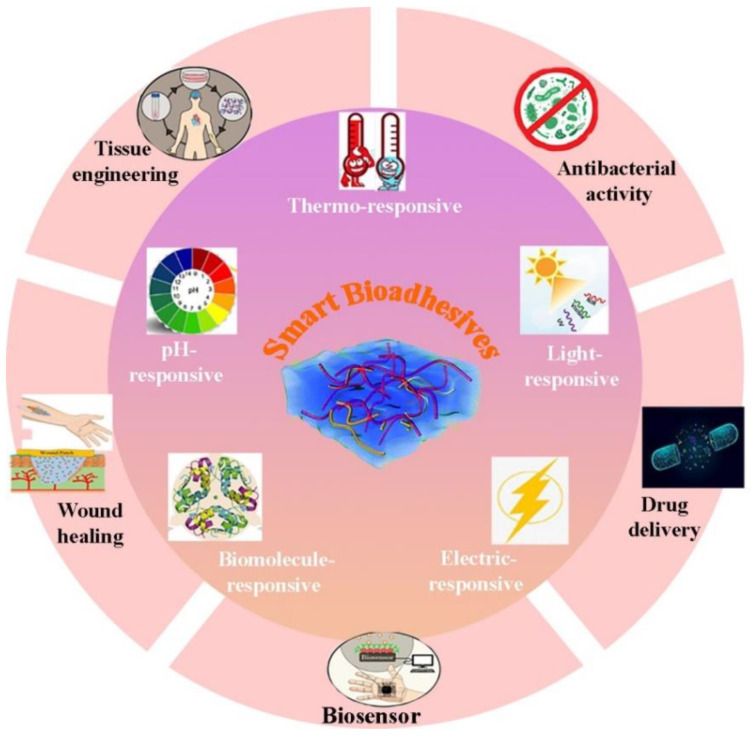
Schematic representation of various types of intelligent bioadhesives applied for different biomedical applications.

Advances in mono bioadhesives have been reviewed elsewhere [3,10],and are not the focus of this review article. Moreover, the history and classification of bioadhesives [5], various types of polymers and composites used as bioadhesives [11], as well as the application of bioadhesive hydrogels for drug delivery (via buccal, transdermal, gastrointestinal, parenteral, vaginal, and rectal routes) [4], wound healing (wound closure, sealing leakage, and immobilization) [12], and biomedical engineering [13] have been discussed by various research groups. In another review, hydrogels with multiple stimulus-responsive mechanisms were introduced, and their applications in emerging biomedical applications were examined [14]. Furthermore, basic background knowledge in designing environment-sensitive hydrogels [15], chemical force microscopy of stimuli-responsive adhesive copolymers [16], and their use as an intelligent carrier in the clinical field have been addressed [17]. In 2018, El-Sherbiny et al. [9] described some synthesis approaches, unique features, and different structures of stimuli-responsive polymers in thin films and nanostructures. Over the years, many disciplinary endeavors have been reported to optimize the functionality of smart bioadhesives and explore new and innovative applications. Nevertheless, investigations are ongoing in the field of smart bioadhesives. Despite extensive studies on stimulus-responsive polymers [18] and hydrogels [19] and many disciplinary attempts to optimize the functionality of smart bioadhesives, according to our knowledge, the development of multifunctional bioadhesives for various biomedical applications has not been adequately explored. In addition, some published review papers have directly or indirectly focused on the biomedical applications of adhesive and non-adhesive hydrogels [20,21,22,23,24], for example, Hwang et al. describe fundamental adhesion mechanisms in the development of multifunctional advanced skin adhesive patches. In comparison with previous review articles, this article provides a comprehensive overview of developed smart adhesives, their limitations, and future directions and challenges for the next generation of smart bioadhesives. In this regard, we will summarize the most recent cutting-edge strategies (years 2015–2021) used for stimuli-sensitive bioadhesives that can respond as external stimuli, self-heal, and remold shapes. First, the principal criteria for bioadhesives and types of smart bioadhesives will be discussed. Hereafter, the applicants of smart bioadhesives in various applications, including tissue engineering, wound healing, and drug delivery, are being studied. Figure 1 schematically presents an overview of these technologies and their applications. Finally, the limitations and challenges of current studies and future directions of smart bioadhesives will be discussed.

## 2. Principal Criteria in Bioadhesive

The adhesion term is defined as the potency of the adhesive to flow, wet the entire surface, and develop specific physicochemical intermolecular forces between the substrates and adhesive [25]. The phenomenon that two materials, one of which should be biological in character, are linked for a long time by interface tailoring is known as bioadhesion [3]. A bioadhesive system aims to make tight contact with the biologic substrate for a long time by interfacial forces. Bioadhesion in biological systems can be divided into class one with attachment between two biological phases, such as platelet aggregation and tissue repair; class two, adhesion between two biological phases (e.g., platelet aggregation and wound healing); and class three, adhesion of a biological degree to an artificial substrate (e.g., cell adhesion to culture dishes and biofilm formation on artificial devices), and adhesion of artificial material to a biological substrate (e.g., adhesion of synthetic hydrogels to soft tissues and adhesion of sealants to dental enamel), which are all examples of bioadhesion in biological systems [26,27]. Irrespective of the type and application, three factors are essential in the design of bioadhesives, including the capability to create powerful interfacial interactions, the ability to maintain cohesive character after curing, and the need to remain biocompatible during bioadhesive service life [5]. In addition to the mentioned characteristics, bioadhesives should have additional properties for specific applications. Bioadhesives should form strong interfacial interactions with tissue. These interfacial bonds can comprise hydrogen bonds, hydrophobic interactions, electrostatic interaction, diffusion, physical interlocking, and chemical cross-linking (such as ester, isocyanate, and aldehyde groups to primary amine) [28]. Nevertheless, the physical interactions poorly act within a high humidity environment because water interferes and may not be impressive in a biological system [29]. In some situations, the incorporation of cross-linking agents into adhesive systems leads to the formation of covalent interactions, such as disulfide cross-linking, Schiff-based chemistry, and enzyme-mediated cross-linking [30]. Therefore, these techniques can be applied to fabricate two and three-dimensional (2D and 3D) networks. However, the development of strong interfacial bonds is not sufficient, and bioadhesives should provide cohesive strength and remain stable for specific times to have adequate support for wound healing [16]. On the other hand, controlling bioadhesives’ hydrophilic and swelling ability is essential for delivering therapeutically active agents in various ways, such as oral and transdermal [4,31,32]. In addition, bioadhesives should be biocompatible to achieve favorable in-vivo results. This means that they do not provide any unpleasant systemic or local effects during deployment and throughout the lifetime of the bioadhesive. In this regard, bioadhesives should not be cytotoxic, allergenic, irritants, or carcinogenic and need to maintain their mechanical integrity with surrounding tissues [33].

Tissue sealants, tissue adhesives, and hemostatic agents are examples of biomedical adhesives. Bioadhesives are categorized into internal and external, depending on the function and compatibility. Internal (intra-corporal) bioadhesives are often applied to repair chronic organ leakages and reduce bleed complications [34]. On the contrary, external bioadhesives are commonly utilized for wound closure and epidermal grafting. Based on the interaction with various organs and tissues, internal bioadhesives are likely to show better biocompatibility, adhesiveness, and strength properties on wet surfaces/environments than external bioadhesives. External bioadhesives are also expected to have a shorter closure time and higher durability than internal bioadhesives [35]. On the other hand, intelligent bioadhesives can also be categorized into three main classes depending on the type of stimuli: chemical (pH, oxidant, and glucose), physical (temperature, light, ultrasound, and pressure), and biological (enzymes, antigen, and ligand) responsive [36,37]. Smart bioadhesives are discussed in the following section.

## 3. Smart Bioadhesives and Their Applications

Smart bioadhesives are attributed to stimuli-responsive compounds with high performances that demonstrate reversible transitions in properties, including solubility, shape, molecular assembly, and surface characteristics in response to a stimulus [38]. Responsive compounds with dynamic properties, including wettability switch, mass transport, and mechanical actuation to inert materials, can have tremendous effects on smart bioadhesives [39]. In general, reversibility in bond association and dissociation aids in the reconstruction of polymeric networks in bioadhesives with stimulus-responsive characteristics. A structurally dynamic material is used in a distinct method for reversible adhesion. The presence of an active bond in structurally dynamic materials allows the material to change one or more properties [40] reversibly. When subjected to a suitable stimulus, the dynamic bond will undergo constant reversible exchange/cleavage, resulting in changes in the material’s properties, such as modulus and viscosity. The bonds stop exchanging when the trigger is removed, and the material returns to its previous state [41]. For example, poly(*N*-isopropylacrylamide) (PNIPAM) is a promising macromolecule in thermo-responsive bioadhesives with a low critical solution temperature (LCST) of about 32 °C. According to the reports, hydrophilic PNIPAM reversibly alters to a hydrophobic state by increasing the external temperature until LCST. So, adhesiveness occurs between the room temperature and the body temperature [42]. Poly (acrylic acid) (PAA) is also a pH-responsive bioadhesive, which can protonate or deprotonate with pH changes. PAA can be swelled through electrostatic repulsion and experience high sorption and release in drug delivery systems [43]. Impressive self-assembly in the liquid state and mass transport in the solid-state can be attained in light-responsive polymers such as azobenzenes that isomerize quickly from one state to another and change size under UV light [44]. Electrochemical-responsive polymers are another smart adhesive group that responds to electric fields by changing their size or shape [45]. More details on the types of smart bioadhesives and their applications are provided in the following section.

### 3.1. Light-Responsive Bioadhesives

**Figure 2:**
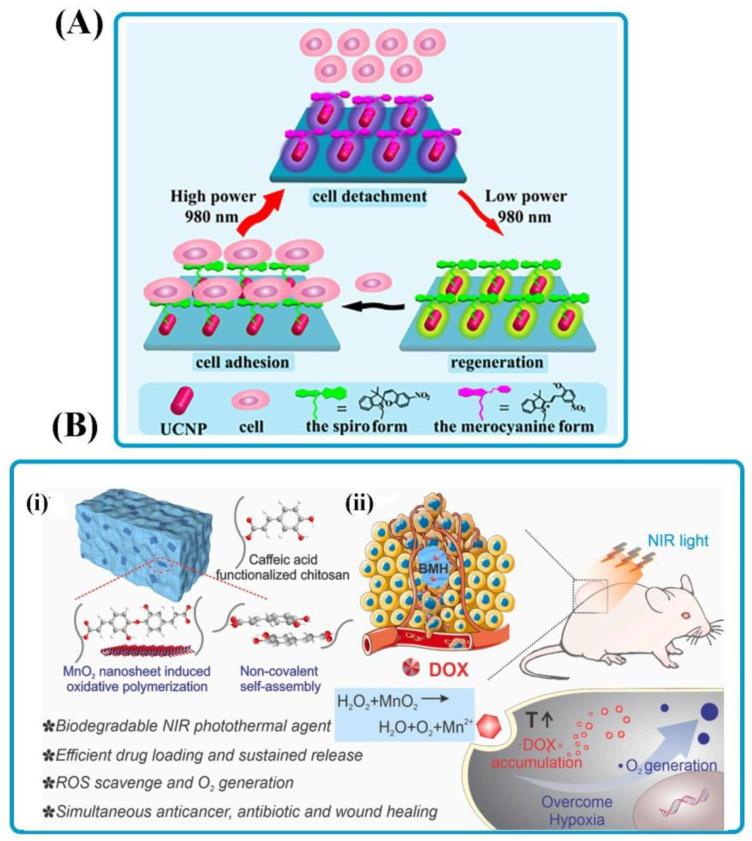
Light-responsive bioadhesives. (**A**) An illustration of SP–UCNP usage as a NIR-triggered photo-switch to modulate cell adhesion/detachment in a non-invasive and reversible manner by adjusting the power density of a laser. Reprinted with permission from Ref. [51]. Copyright 2015, ACS Publications. (**B**) (**i**) Schematic illustration of BMH hydrogel’s composition and structure for simultaneous anti-cancer treatment and MDR bacteria-infected scar tissue. (**ii**) The nanostructure of BMH hydrogel effectively increased chemotherapy by enhancing O_2_ generation via breaking endogenous H_2_O_2_ and enhancing intracellular buildup of DOX by PTT Reprinted with permission from Ref. [53]. Copyright 2020, Elsevier.

Light-responsive smart adhesives as noninvasive tools to regulate cell adhesion can be applied to tissue engineering, cell diagnostics, and medicine. The physicochemical behaviors of photosensitive molecules are altered or degraded in response to light irradiation with suitable wavelength and intensity [46,47]. Table 1 summarizes light-responsive bioadhesives trouped by stimulus responses and contains information about the inspiration and application. The light-responsiveness of O-nitrobenzyl was first cited by Ciamician and Silber about a century ago [48]. UV radiation activates most photochemical processes, such as acrylate polymerization, thiol-ene reaction, nitrobenzyl, and spiropyran groups. However, UV light-induced injury to biological specimens and live organs may restrict its use in-vitro and in-vivo because of intrinsic cytotoxicity and poor tissue penetration [49]. Photo-activation with near-infrared (NIR) light due to neglectable phototoxicity, easy access, clean, inexpensive, and sufficient penetration into the tissue can be considerable [50]. Li et al. [51] utilized spiropyran (SP) conjugated multi-shell upconversion nanoparticles (UCNPs) for adjusting cell adhesion/detachment reversibly and noninvasively. The UCNPs are ceramic lattices incorporated with trivalent lanthanide ions that could convert NIR light to UV radiation and activate photochemical processes on request. High-power and low-power NIR treatments were used to activate ring-opening and ring-closing procedures, respectively (Figure 2A). Such conversions caused the relation between SP and the cellular protein surface to be replaceable, resulting in reversible cell adhesion and detachment. Bian et al. [52] synthesized a reversible visible-light-responsive biofunctional surface by interacting the host–guest of azobenzene derivatized polycation/polyanion on a cyclodextrin (CD)-terminated substrate for switching from antibacterial to bioadhesion. They showed that the polyanions with COO^−^ groups provided bioadhesive properties, while the azobenzene functionalized polycations with quaternary ammonium groups had vigorous antibacterial activity. They could be switched by alternate assembly when exposed to visible light. Light-responsive bio-inspired MnO_2_ hybrid (BMH) bioadhesives were employed in a research study for efficient melanoma photo-thermo-chemotherapy and multidrug-resistant (MDR) bacteria-contaminated healing of wounds(Figure 2B) [53]. As one of the “light-responsive” materials, MnO_2_ nanosheet was produced to induce spatial and temporal controlled hyperthermia for further photothermal therapy. Furthermore, the two-dimensional nanosheets could be perfect bioactive molecule delivery carriers, allowing potent synergistic treatments for cancer and wound healing due to their high surface area and high binding energy via electrostatic and polar interactions. Based on the results, enhancing the local access to oxygen increased the cellular toxicity of doxorubicin (DOX) versus melanoma.

Although light-responsive bioadhesives possess many advantages, such as minor damage to cells, remote modulation, and high controllability of stimulus, they still have several limitations to being converted into medical products, including the incapacity of light sources to penetrate tissue [59], the use of UV light as a non-biofriendly source [60], and weak mechanical strength [61]. Many light-responsive bioadhesives require complex synthesis techniques that restrict their potential to be developed. Though NIR-triggered agents are more popular than UV-triggered agents, no investigation studied the effect of NIR-triggered agents on deep tissues. Only a few studies have been conducted on superficial disease models. In addition, due to their lower efficiency, these systems require a longer exposure time to have a therapeutic impact [62]. The unwanted extreme warmth may injure the surrounding healthy cells as a result of the unwanted extreme warmth. Furthermore, most of these studies were conducted in-vitro, and it is essential to continue development and confirm results in-vivo.

### 3.2. Thermo-Responsive Bioadhesives

The temperature is of great interest among external stimuli because of its broad application, effortless control, and capability to use in-vitro and in-vivo states [63]. As a minimally invasive technique, thermo-sensitive bioadhesives show conformational changes in response to temperature stimuli, particularly near-physiological human body temperature, to generate interim polymer chain cross-linking via multiple physical interactions [64]. An ideal thermo-responsive system is a polymer solution with low viscosity at ambient temperature that, after injecting into target sites, changes into a gel at body temperature. Some studies focused on thermo-responsive bioadhesives are listed in Table 2.

**Figure 3:**
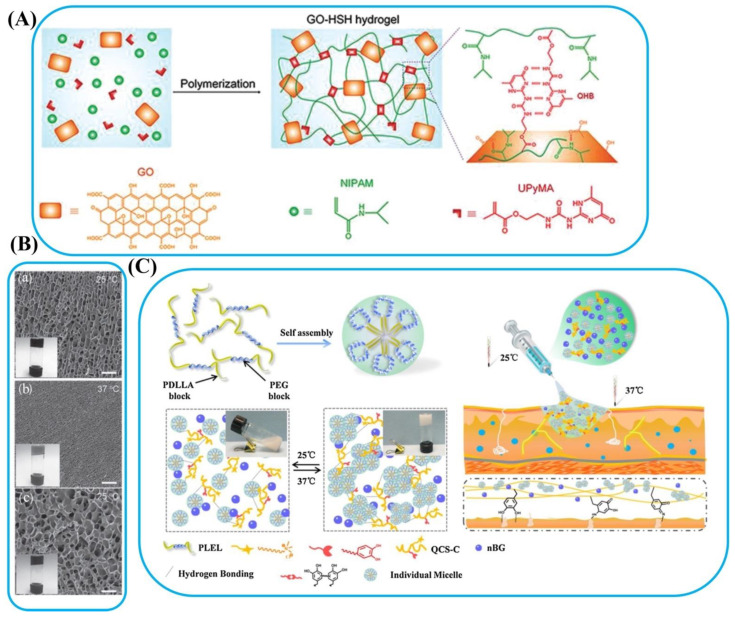
Thermal-responsive bioadhesives. (**A**) Schematic models of the synthesis of supramolecular hydrogel (HSH) containing GO, NIPAM, and UPy ethyl methacrylate monomer. (**B**) SEM images of a bioadhesive in both states of hydration and dehydration: (**a**) at 25 °C, (**b**) dehydrated at 37 °C above the LCST, and (**c**) restored to the hydrate conditionat 25 °C. Reprinted with permission from Ref. [78]. Copyright 2017, Wiley-VCH. (**C**) Schematic diagram of thermo-sensitive injectable PLEL-nano bioactive glass-QCS-C composite hydrogel for wound healing. Reprinted with permission from Ref. [70]. Copyright 2020, Elsevier.

Polymers are classified into two categories [68]. The first case is LCST, which is insoluble above its critical temperature. The second case is the upper critical solution temperature (UCST), which precipitated and underwent a phase shift in its critical temperature, the temperature at which the polymers keep miscible in solution. At the same time, phase separation occurs when the temperature rises over the critical value, which is called “negative temperature-sensitive polymers” in LCST materials (e.g., PNIPAM, gelatin, and carrageenan) [76]. In contrast, UCST materials are known as “positive temperature-sensitive polymers”. They are miscible at room temperature, while their solubility diminishes when the temperature drops below the critical value, causing phase separation. Examples include (acrylamide-co-butyl methacrylate), PAA, and polyacrylamide (PAAm) [77]. The thermo-sensitive microstructure changes of a supramolecular hydrogel bioadhesive containing ureidopyrimidinone (UPy) and PNIPAM (Figure 3A) were investigated by scanning electron microscope (SEM) images (Figure 3B) [78]. This bioadhesive displayed large pores at the 25 °C (approximately 3.4 µm) while the size of the pores decreased to around 0.82 µm at 37 °C. Interestingly, the pores could regain their primary size (about 4.0 µm) by turning back the temperature to 25 °C. This suggested that the macromolecule chains became hydrophilic at a lower temperature than the LCST and may generate bigger pores during the lyophilization process. In contrast, the polymer chains were dehydrated and collapsed at a temperature higher than LCST, and the size of pores became smaller. This bioadhesive was a good candidate for drug delivery application. Zheng et al. [70] designed a bioadhesive based on quaternized chitosan (QCS-C) embedded into poly(d,l lactide)-poly(ethylene glycol)-poly(d,l-lactide) (PLEL) for wound healing. In the below LCST, the PLEL polymer was a random coil unimer (Figure 3C). With the increase in temperature above LCST, the PLEL structure changed to a micelle because of the hydrophilic poly(ethylene glycol) chain (outer shell) and the hydrophobic poly(d,l-lactide) chain (inner core). The presence of QCS-C could be effective in decreasing sol–gel transition temperature. In addition, the rheological property of the bioadhesive indicated that it could flow freely below the gel point and was fully suitable for in situ injection. As the temperature increased from 33 to 40 °C, the storage modulus approached the loss modulus, indicating a semisolid property. As a result, the human body’s temperature may be ideal for therapeutic wound management. Zhang et al. [71] prepared a thermos-responsive bioadhesive by simply combining galactose modified xyloglucan (mXG) and hydroxybutyl chitosan (HBC). The obtained bioadhesive as a cytocompatible and hemocompatible hydrogel prevented repeated adhesion after adhesiolysis, enhanced wound healing, and reduced tissue injury.

**Figure 4:**
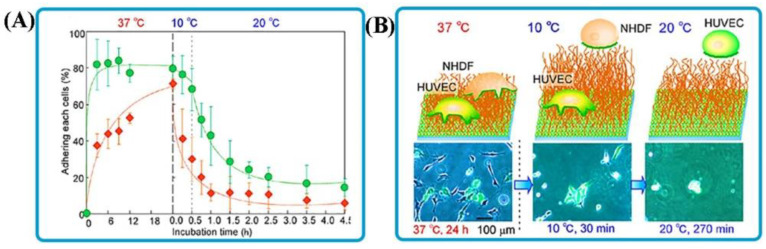
Smart bioadhesives for tissue engineering. (**A**) Diagram of adhesion on and detachment of green fluorescent protein (GFP)-HUVECs and NHDFs from IPB-5 in culture medium. NHDFs and GFP-HUVECs are represented as orange squares and green circles, respectively. Cell adhesion was carried out at 37 °C for 24 h; after which the cells were incubated for 30 min at 10 °C, followed by a recovery period at 20 °C. (**B**) Morphology of GFP-HUVECs and NHDFs on and detachment from IPB-5. Reprinted with permission from Ref. [72]. Copyright 2013, ACS Publications.

Comprehensive studies have focused on applying thermo-sensitive bioadhesives for tissue engineering and in vitro transplantable tissues. In tissue engineering and regenerative medicine, intelligent bioadhesives can be employed as injection systems to transfer growth factors and cell stimuli-responsive surfaces to regulate cell adherence or penetration [79]. For instance, Moreira et al. created a bioactive thermogelling chitosan-based injection of bioadhesive hydrogel for bone regeneration. Recently, regenerative medicines with the ability for cell culture to remedy the lost functions of organs and tissue have been becoming promising treatments. To form transplantable tissues invitro, selecting a cell separation method with enough purity and function after dissociation is interesting. In this regard, the separated cells using the thermo-responsive adhesive brush have shown high function [80]. Even though this process requires a relatively long time, cell purification is not needed for constructing tissues. Moreover, separated cells using this polymer show good function without correction of the cell surface, which is significant for manufactured tissue transplantation.Furthermore, the separation can be accomplished simply by changing the external temperature of the adhesive brush surfaces that have been created [81]. Polymer brushes are unique macromolecular structures with a dense array of polymer chains immobilized on a surface or interface by one of their end chains. These structures have promising applications for stimuli-responsive and cell adhesive surfaces [82]. In the study by Nagase et al. [72], thermo-responsive copolymer bioadhesives were developed by copolymerizing butyl methacrylate (BMA) into PIPAAm. The adhesion characteristics of copolymer brush surfaces at 37 °C and detachment at 20 or 10 °C were confirmed for human umbilical vein endothelial cells (HUVECs) and normal human dermal fibroblasts (NHDFs), respectively (Figure 4A,B).

To investigate bone tissue repair, Saravanan et al. [83] developed a thermosensitive chitosan/glycerophosphate adhesive hydrogel containing graphene oxide (GO) with the applicability of injectable. They found that the inclusion of GO into the matrix significantly improved swelling and protein adsorption ability. They further concluded that a GO-containing chitosan/GP hydrogel possessed the ability to produce osteogenic differentiation of mesenchymal stem cells (MSCs), making it appropriate for bone tissue engineering.

**Figure 5:**
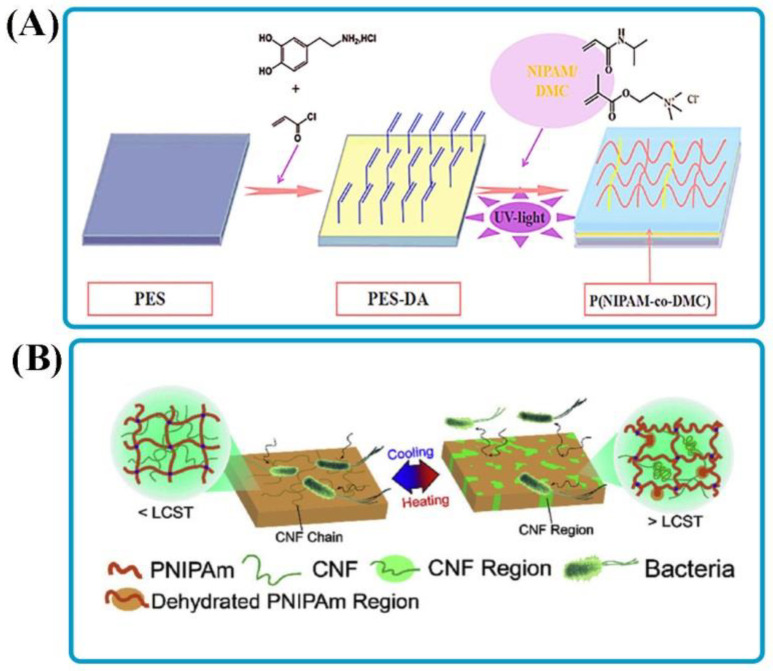
Smart bioadhesives for antibacterial activity. (**A**) A schematic illustration of modification procedure of PES membranes by ene-functionalized dopamine to form an adhesive layer and then attaching it onto the membranes via photo-induced surface cross-linking copolymerization. Reprinted with permission from Ref. [84]. Copyright 2018, Wiley-VCH. (**B**) Schematic of the effect of temperature on microorganism’s growth and adhesion for PNIPAm/CNF hydrogels. Reprinted with permission from Ref. [86]. Copyright 2020, Elsevier.

The induction of antibacterial properties is another promising application of thermal-responsive adhesives. The mechanism of antibacterial surfaces depending on bactericidal agents is classified into releasing-based and contacting-based bactericidal agents [84]. In the first case, biocides are usually incorporated or preloaded into a matrix and then released into the surroundings to kill the bacteria (e.g., releasing drugs to decrease infection in sores). Bioadhesives containing quaternary ammonium are extensively utilized for contacting-based mechanisms for the second group. It is related to the low toxicity, excellent cell membrane infiltration character, extended residence time, environmental constancy, and biological activity of ammonium [85]. For example, dopamine (DA) as an anchoring site was loaded into the polyethersulfone (PES) membrane surface to develop an adhesive layer, then the adhesive was stuck onto the membrane by using photoinduced cross-linking copolymerization of methacryloxyethyltrimethyl ammonium chloride (DMC) and NIPAAm (Figure 5A) [84]. The results showed that the quaternary ammonium salts in the hydrogel film could lead to the destruction of the adhering bacteria. On the other hand, the dead bacteria detached from the surface by decreasing the temperature below the LCST of PNIPAM. In addition, the clotting test revealed that the changed surfaces improved blood compatibility and prevented hemolysis. In other work, 3D printable thermo-responsive PNIPAM/cellulose nanofibrils (CNFs) were developed to provide a new platform for regulating LCST properties and tuning bioadhesive behaviors [86]. In response to temperature, the hydrogel system containing 2% CNF had exchangeable bioadhesion. Above the LCST, the adhesion of the PNIPAm/CNF hydrogels to bacteria was stronger. It could be related to the wholly extended CNF, which made a semi-interpenetrating polymer with PNIPAm. The bioadhesive was severely weakened at 40 °C. The CNF chains were divided into small separate sections, considerably reducing the bacteria-CNF contact area. Therefore, temperature control might be utilized to keep or release bacteria that have developed on the hydrogel surface (Figure 5B).

Despite advantages such as easy accessibility, low side effects, stability of drugs, etc., thermo-responsive materials have disadvantages such as poor mechanical strength, limitation in drug loading ability [87,88], and uncontrollable on/off state of actuation [89], which need more studies.

### 3.3. pH-Responsive Bioadhesives

Ionic polymers are commonly used in pH-responsive adhesives. Protonation or deprotonation of ionic side chains in these polymers can cause swelling of polymer backbones due to electrostatic repulsion [90]. pH-responsive materials can be divided into anionic and cationic bioadhesives based on the pendant group in the polymer chains [91]. When the adhesive’s acid dissociation constant (pK_a_) is lower than the pH of the surrounding aqueous solution, the swelling/deswelling behavior of anionic materials is triggered by osmotic pressure. In contrast, the cationic materials that contain donor electron groups such as amine become protonated and swelled in an aqueous solution with a lower pH (<pK_a_) [14]. For a better understanding, some polymers’ swelling and shrinking behavior, such as chitosan, is referred to as external and environmental pH [92]. At lower pH, the protonation of the amine group of chitosan generated electrostatic repulsion, allowing polymer chains to extend and interact with water molecules more efficiently, thereby enabling water solubility. The amine group is deprotonated when the pH rises, implying no net charge [93]. Further, the amine groups destroy the chitosan structure and decrease the water solubility. As a result, the pK_a_ value is effective in the water solubility of some pH-responsive polymers. Pores are typically produced in other polymers with pH-dependent solubility for more specialized pH-sensitive uses. The waterinsolubility of these polymers at low pH (e.g., in the stomach) and their solubility at higher pH (e.g., in the small and large intestines) causes materials to leach out and form a porous and permeable film. In bioadhesives, synthetic polymers such as PAA, proteins, and polysaccharides are commonly classified as pH-sensitive polymers [5,94]. According to studies, diseasedcaries in people’s mouths fluctuate between 4.5 and 6. Therefore, dual adhesive membranes and oral drugs can be applied to protect from oral infections and oral tissue regeneration. Table 3 summarizes different types of pH-responsive bioadhesives and their applications.

**Figure 6:**
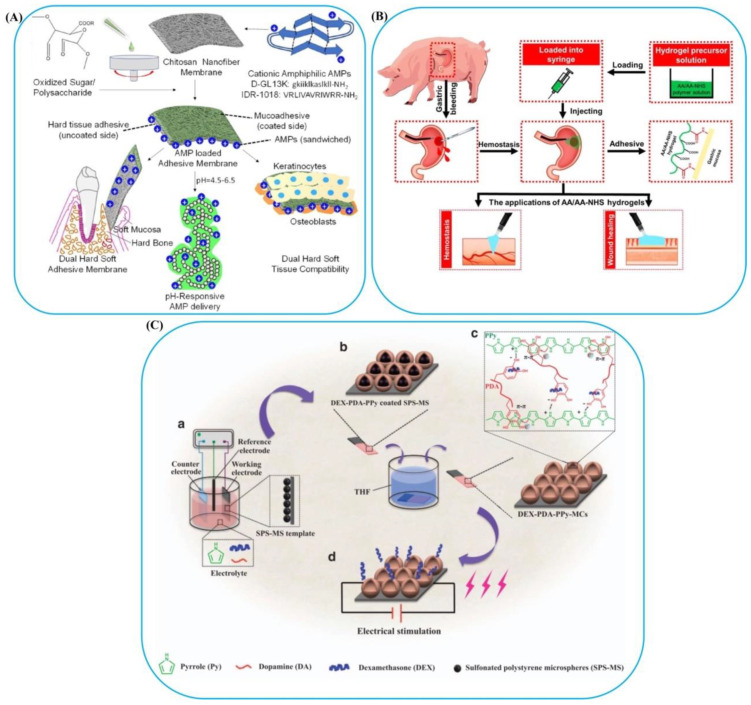
(**A**) pH-responsive bioadhesives. A scheme of the preparation stages of oral tissue adhesive membranes coated with AMP and pH-responsive release of AMP to acidogenic oral biofilm. Reprinted with permission from Ref. [101]. Copyright 2020, ACS Publications. (**B**) The use of AA/AA-NHS bioadhesive hydrogels for wound healing and blood clotting. Reprinted with permission from Ref. [104]. Copyright 2021, Springer Nature. (**C**) Electro-responsive bioadhesives: An illustration of the procedure for preparation of dexamethasone-loaded PDA–PPyMCs, (**a**) process of electrochemical deposition, (**b**) eliminating the sulfonated polystyrene microspheres template by tetrahydrofuran etching, (**c**) hydrogen bonding, and π–π interactions between PDA and PPy, and (**d**) drug delivery by electrical stimulation. Reprinted with permission from Ref. [107]. Copyright 2017, Springer Nature.

For pH-controlled delivery of antimicrobial peptides (AMP) into the oral cavity, Boda and coworkers [101] prepared bioadhesive membranes combined with chitosan and pectin derivatives with dual adherence to soft and hard tissue surfaces (Figure 6A). Pure chitosan membranes indicated suitable adhesion to enamel tissue/hard, whereas the presence of oxidized pectin can be an effective way to increase mucoadhesion. One of the drawbacks of this work was that the effect of pH on the adhesive qualities of membranes was not studied. Yadav et al. [103] developed pH-sensitive adhesive for antibacterial applications. They learned how to brush dispersity and thickness affected the initial attachment and future detachment of *Staphylococcus epidermidis* bacteria to a pH-responsive PAA brush system. With increasing pH value, the instinct properties of PAA changed from neutral, hydrophobic, and dried up to negatively charged, hydrophilic, and swollen. Switching from pH=4 to 9 also removed microorganisms from the brush surface. Based on the results, an optimal thickness of 13 up to 18 nm was recognized for maximizing microorganism detachment on the PAA brushes at pH 4. The brush dispersion did not affect bacterial adhesion. Recently, a series of injectable pH-responsive self-healing bioadhesives have been produced by radical polymerization of acryloyl-6-aminocaproic acid (AA) and AA-g-N-hydroxysuccinimide (AA-NHS) for wound healing applications [104]. The good hemostatic performance, histomorphological evaluations, and wound healing results demonstrated the therapeutic efficacy of the AA/AA-NHS hydrogel in a swine gastric hemorrhage/wound model (Figure 6B).

Despite the reality that pH-responsive bioadhesives have demonstrated wide applications in medicine, there are still issues with the evolution of a bioadhesive, which can behave in favorable procedures under basic and acidic situations. The swelling property is necessary for bioadhesives because liquid absorption is critical during tissue regeneration [105]. However, some adhesives lose their mechanical strength due to solution uptake. On the other hand, the pH value of the media pH may differ depending on the intensity of the complaint or the type of damaged tissues, making it difficult to maintain the bioadhesive’s adhesion capabilities during the therapy process [106]. Furthermore, the actions of pH-sensitive bioadhesives might be initiated during administration or use, rendering these systems susceptible to off-target distribution.

### 3.4. Electromagnetic-Responsive Bioadhesives

Field-responsive polymers can be exploited in applying sonic, magnetic, electric, and electromagnetic fields. An electric field’s changing geometrical shapes and sizes can be depicted as a synergy of coulombic, electrophoretic, and electroosmotic interactions [47]. Electro-responsive polymers are classified into two groups: ionic and dielectric [108]. Various types of electro-responsive bioadhesives and their applications are given in Table 4. The first group is known as conducting polymers, in which response to an electric field leads to the mobility of free ions and a change in the local concentration of ions in solution or within the material [109]. The migration of ions in an electric field can cause the asymmetric distribution of charged ions, the formation of a concentration gradient of ions, the generation of an osmotic pressure difference, and finally, the production of a swollen hydrogel. On the other hand, the second group includes dielectric elastomers and electrostrictive polymers created by electrostatic (coulombic) forces [110]. Polydopamine–polypyrrolemicrocapsules (PDA–PPyMCs), as electro-responsive and conductive polymers, have been synthesized on titanium electrodes that can release medications locally and accurately [107]. The preparation steps of the electro-responsive system are described in Figure 6C. Based on the results, the adhesion strength of the PDA–PPy for reacting with various substrates was enhanced by increasing the PDA amount. Also, PDA–PPyMCs presented an excellent ability to attach to cells and drug-loading due to strong cell affinity, porous form, electro-responsivity, and good conductivity. Thismeans they could be used as a conductive substrate to transfer electrical impulses to stimulate cell action. However, precise control over the magnitude and duration of electric current provided a unique advantage to electro-responsive bioadhesives. Moreover, their applications have drawbacks, such as wired and bulky instruments [111]. Furthermore, they need to implant electrodes in the bioadhesive matrix, which determines their applications for topical or subdermal implants.

### 3.5. Biomolecule-Responsive Bioadhesive

Sensitive bioadhesives to biomolecule amounts can activate the localized therapeutic drug release to mimic the short- and long-term molecular regulatory processes seen in tissues at the cellular level. Biomolecule responsive bioadhesives have been highly regarded for their structural transition in response to the main target biomolecule [119]. For instance, glucose-responsive adhesives can indicate structural changes in reaction to the glucose amount in diabetes disease. Insulin injections may possess various problems, such as a long treatment period and diet restrictions, which present an alternative therapy way with the capability of immediate responses to blood glucose levels; safe and continual administration seems essential [17]. Types of electro-responsive bioadhesives and their applications are listed in Table 5. Zhou et al. [120] developed a stimulus-sensitive turnover method using a bioadhesive oral delivery nanoparticle system coupled with glucose oxidase (GOx) and insulin as an intelligent glucose-responsive switch. The L-cysteine–alginate in glucose-responsive nanoparticles with a suitable weight ratio of 2:1 showed good encapsulation efficiency, bioadhesion, and pH stability, which are favorable for oral delivery. In-vitro studies revealed that glucose-responsive nanoparticles switch insulin release behavior “ON” in response to a hyperglycemic condition by catalysis of GOx and “OFF” in response to normal blood glucose levels. Despite promising features, obstacles such as poor stability in various environmental conditions, unfavorable behavior at physiological pH, poor glucose selectivity, and slow response rate raise concerns about the clinical usage of glucose-responsive bioadhesives [121].

### 3.6. Multi-Responsive Bioadhesives

**Figure 7:**
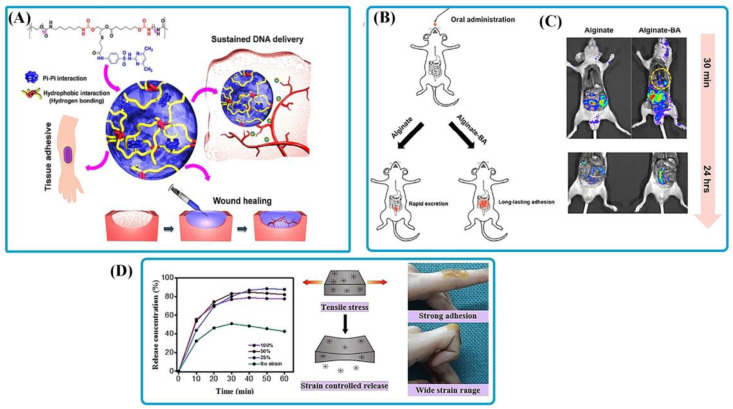
Multi-responsive bioadhesives. (**A**) Schematic of transition of sol-to-gel phase in PEG–PSMEU bioadhesives and their biomedical application in wound healing. Reprinted with permission from Ref. [128]. Copyright 2018, ACS Publications. (**B**) Schematic of oral delivery of alginate and alginate-BA to mice. (**C**) BALB/c mice were given rhodamine B isothiocyanate–dextran plus alginate (left) and alginate-BA (right) solutions and killed after 30 min and 24 h. Reprinted with permission from Ref. [129]. Copyright 2018, ACS Publications. (**D**) Pattern of strain-controlled release related to GA hydrogel adhesive at different strain percentages and strong adhesion on human skin with stretching. Reprinted with permission from Ref. [130]. Copyright 2020, Elsevier.

Multi-responsive bioadhesives have been created to develop multifunctional bioadhesives for different biomedical applications [125]. These bioadhesives are specifically attractive for drug delivery applications. Recent advances in intelligent drug delivery adhesive carriers have great promise. They provide a way to promote formulations tailored to drug delivery systems and the release of drug control based on stimuli responses [126]. An intelligent drug delivery system can release an active chemical at the proper place and at a rate that adjusts in response to disease progression [127].Some of the multi-responsive bioadhesives and their applications are provided in Table 6. Le et al. [128] synthesized pH and temperature-sensitive injectable bioadhesives of poly (sulfamethazine-ester-urethane) (PSMEU) and poly(ethylene glycol) (PEG) by in-situ developing injectable hydrogelators (Figure 7A). Although PEG–PSMEU bioadhesive was free-flowing at ambient temperature, it quickly became a gel when exposed to body physiological conditions (pH 7.4 and 37 °C). These bioadhesives could promote skin wound repair due to their superior adhesive, bioresorbable, and mechanical characteristics. In addition, hypodermic implantation of PEG–PSMEU repaired the damaged skin and led to wound healing without an inflammatory response. In their research, Lee et al. [129] indicated that alginate–boronic acid hydrogel could be orally administrated in drug delivery systems because of its tolerance to high acidic conditions in the stomach (Figure 7B). In addition, the adhesive nature of alginate-BA could increase the residual time in the body. For the first 30 min, a clear fluorescent signal was observed in the esophagus region for alginate-BA (Figure 7C, right panel, yellow circle). It was related to alginate-BA’s gelation in the esophagus region (pH upshifts to 7.4). On the other hand, alginate was wiped off, and no fluorescence was noticed when administered orally (top, left panel). The alginate-BA hydrogel led to strong fluorescent signals (e.g., red spots) in the colon after 24 h. However, alginate alone had weak dispersive signals of residual fluorescence (bottom, left panel). Abebe et al. [130] synthesized a self-adhesive hydrogel based on modified alginate with gallic acid (GA) and in-situ polymerization of polyacrylic acid for pH and strain-responsive transdermal delivery. According to the findings, stretching led to an increase in the release rate, but strain percentage had the opposite impact. As demonstrated in Figure 7D, strain percentage of 100% resulted in increased release within the first 10 min, but the releasing pattern reversed after 20 min at pH = 5.5. In 2019, a triple stimuli-responsive hydrogel with self-adhesive, self-healing capabilities was developed using *N*, *N*-diethylacrylamide (as a thermo-sensitive part), PDA (as a NIR light-sensitive part), and acrylic acid (as a pH-sensitive part) [131]. The resulting bioadhesive was extensively employed in wound dressings and wearable technology. Furthermore, the hydrogel with an optimum mass fraction of 0.4 wt.% of PDA was for the removal of methylene blue, with a maximum adsorption capacity of 305.4 mg/g.

Ionic liquids (ILs) are a type of organic salt that is made up of cation–anion pairs of an organic ion and an inorganic counterion, in which the cationic or anionic part is a relatively large organic moiety [144]. These cations and anions affect their properties in these compositions, including polarity, electroconductivity, viscosity, and volume. Consequently, ILs are ionresponsive making them attractive for various applications, including multi-responsive adhesives [145]. For example, dual-cross-linked ionohydrogelhas been developed using IL binary solvent system [142]. The IL incorporated adhesive hydrogel revealed admirable mechanical characteristics, transparency, high ionic conductivity making it promising for flexible ionotronic adhesive devices. Kuddushi et al. [141] also developed a stimuli-responsive and self-healable bioadhesive based on an ester-functionalized IL. Results demonstrated that the hydrogel was responsive to intracellular biological stimuli, including acidic pH of cancerous cells and temperature, making it promising for the controlled release of anticancer drugs. In addition, the morphology of hydrogel was changed by changing the shape and size of the gelator. In another interesting study, microwave-responsive adhesives were developed using simple mixing of acrylic adhesives with ionic liquids [146]. Fast response to microwave irradiation was reported via local heating of the IL. This response resulted in adhesive failure in less than 30 s. One of the main applications of ILs based adhesives is sensing [140]. In a recent study, a self-healable and ultrastrong adhesive ionogel was developed for multifunctional strain sensors. Li et al. [147] prepared a polysiloxane-supported ionogel by locking ionic 1-ethyl-3-methylimidazolium bis(trifluoromethylsulfonyl)imide ([EMIM][Tf2N]), into poly(aminopropylmethylsiloxane) (PAPMS) grafted with [2-(methacryloyloxy)ethyl] trimethylammonium chloride (METAC). Due to its adhesive behavior and high ionic conductivity, the obtained ionogelwas promising for flexible electronic devices such as sensors. In addition, Yu et al. [148] fabricated an adhesive hydrogel by multiple cross-linking between a PIL and k-carrageenan. The results indicate that the addition of PIL has a great influence on the adhesion strength of hydrogels. It can be due to interactions with charged groups or polar groups through ion–dipole and dipole–dipole interactions. Due to having suitable electrochemical performance, high mechanical stability, and strain sensitivity, these conductive adhesives can be appropriate for wearable strain sensors and the monitoring of human health [149].

## 4. Clinical Applications of Stimuli-Responsive Bioadhesives

Intelligent adhesives have been widely applied in biomedical applications, including tissue engineering, drug delivery, epidermal sensors, tissue sealants, and wound healing. The stimuli-responsive bioadhesives can be developed to regulate their adhesiveness based on applied stimulations. After the wound is healed, the bioadhesive must be removed from the wound closure process [150,151]. However, there is a risk of injuring tissue and triggering pain for patients during the removal process. The ability to modulate adhesiveness on-demand with stimuli-responsive bioadhesives allows the bioadhesive to be removed from the wound site without causing any damage or pain [13]. When the intelligent bioadhesive is used to adhere to dynamic tissues, it is subjected to periodic external stresses. The bioadhesive may suffer irreversible physical damage as a result. Self-healing functionality is used as an efficient technique to assure the stability of bioadhesives in dynamic tissue settings. After the physical injury, self-healing bioadhesives repair their mechanical structure while keeping their original characteristics [152]. The stimuli-responsive bioadhesive can be programmed to deliver therapeutic medicines on demand. Due to the dynamic nature of the tissue healing process, temporal management of tissue is required for more effective tissue repair with fewer adverse outcomes. The stimuli-responsive bioadhesives allow for control by releasing antibiotics and therapeutic medicines at the right time to promote cellular differentiation, tissue-specific gene expression, and native tissue healing [153].

Internal body tissues, including bone, heart, nerve, kidney, nerve, and muscle, can also benefit from stimuli-responsive bioadhesives [34]. These interior tissues are moister and rougher compared to the skin tissue environment. The internal tissue environment is intricate and dynamic according to numerous biochemical parameters. As a result, implementing controlled responsiveness can be difficult. Another critical problem is creating stimuli-responsive bioadhesives that are stable in vivo for an extended time [154]. Furthermore, because internal tissue procedures inevitably necessitate incisions, a protracted adhesion process increases the risk of bacterial infection, inflammatory reactions, and tissue damage. Biocompatible bioadhesives with low cytotoxicity have demonstrated an essential role in this field.

Contact between electrodes and tissues is required to obtain electrical biosignals from the body. The touched electrode will experience dynamic motions in epidermal and interior tissues [155]. This might result in mechanical deformations such as stretching, compression, and bending during contact. Internal tissues have a stricter environment for electrode–tissue contact due to muscle contractions from gastrointestinal peristalsis, pulmonary cycles, and heart muscles. The electrodes may now be strongly retained on the tissue surface due to the invention of conductive smart bioadhesives. Furthermore, flexible bioadhesives provide for conformal contact between the electrode and the tissue, allowing direct electrical signal delivery [156]. The clinical applications of smart bioadhesives can be summarized and grouped in Table 7 as follows.

### 4.1. Wound Healing of Soft and Hard Tissues

One of the most common uses of smart bioadhesives is wound healing. For many years, wound closure has been carried out with wires, sutures, and staples [166]. Nevertheless, concerns about the sign of scar, secondary damage, wicking-induced infection, slowed wound healing, and complicated postoperative care has limited their applications. Smart bioadhesives have become more popular because people are more concerned with their physical appearance [167]. Zhao et al. [157] created a new stimulus-responsive bioadhesive made up of a prepolymer of poly(glycerol sebacate)-co-poly(ethylene glycol)-g-catechol. Three incisions (2 cm) were made on the rats’ backs to evaluate their capacity to close wounds. The smart bioadhesive-treated group had higher fibroblast recruitment and proliferation, as well as less inflammatory infiltration, on the seventh day after surgery. Liang et al. [158] fabricated a smart bioadhesive through dual-dynamic bond cross-linking between Fe, protocatechualdehyde containing catechol and aldehyde groups and quaternized chitosan. A full-thickness incision model was used to examine the wound closure’s efficacy. On day 7 post-surgery, the sealed incision treated with the bioadhesive exhibited complete epidermis and dermis structures and higher collagen deposition levels than the control group, and the incision closed with surgical sutures. Treatment for wounds of brittle and hard tissues is another type of wound healing where smart bioadhesives are beneficial [168]. Bioadhesives, especially for small fragments of bone, are a quick and easy way to repair damaged portions of hard tissues. The lack of fixation of the small fragments of bone typically results in bone resorption, which can lead to deformation of the bone union, bone movement, and nonunion [169]. Accordingly, Yan et al. [112] developed an electrically conducting bioadhesive to attach tiny bone fragments in comminuted bone fractures. Aniline tetramer and dopamine were added to the system to enhance the cell adhesion, proliferation, and osteogenic differentiation of MC3T3-E1 cells.

### 4.2. Drug Delivery

The recent innovations in smart drug carrier systems seem promising, as they supply a means to promote formulations of targeted drug delivery systems, and drug release control based on stimuli response [170]. Smart bioadhesives in delivery have an advantage over typical hydrogel delivery systems in that they can fix delivered objects on the site. Mucoadhesion is effective in enhancing the bioavailability of poorly absorbed drugs by lengthening their residence time in the gastrointestinal tract, resulting in lower doses and dosage frequency [159,171]. Yan et al. [160] prepared adhesive hydrogel with pH, temperature, and NIR light-responsive behavior for use in controlled release systems. Zhu et al. fabricated a stimuli-responsive bioadhesive by incorporating graphene aerogel into a PNIPAM network with incorporated PDA nanoparticles. The NIR controllability of the bioadhesive for the DOX release was excellent. When the hydrogels were exposed to a NIR laser for one minute, DOX was released, and the amount released rose dramatically. After the laser was turned off, no more drug release was recorded. Smart bioadhesives can also be an important topic in both agricultural and environmental chemistry. For example, the Ca–alginate/PNIPAm-based photothermal adhesive was designed to control the release of imidacloprid (IMI) by sunlight [172]. Researchers showed that the accumulative release percentage of IMI was about 29.8% at 15 °C and increased to about 60.4% at 40 °C. Smart bioadhesives can also be loaded with cells and growth factors. Using a suitable scaffold biomaterial as a cell transport vehicle can create a favorable microenvironment for extending cell survival [173,174].

### 4.3. Leak Sealants in Medical

A common complication of surgeries and injuries is leakage. Headaches, meningitis, and seizures can result from cerebrospinal fluid leaks caused by traumas or brain and sinus surgery [175]. Gastric fluid leakage, common during surgical operations, can result in infection and significant tissue destruction [176]. As a result, leakage control is critical in lowering operation risks, complications, and costs. Tissue sealants, also known as smart bioadhesives for leakage prevention, have piqued the interest of researchers and have showed tremendous promise in the clinic. Blacklow et al. created a thermo-responsive bioadhesive to speed wound healing, contracted at body temperature. Bioadhesive dressings could help heal wounds in other epithelial tissues such as the gut, lung, and liver. Bleeding is one of the most common side effects caused by surgical procedures, injury, diseases, and medications [177]. Hemostasis sealants are widely accessible on the market. However, they have separate limits. Chang et al. presented a hemostatic photo-responsive bioadhesive based on gelatin methacryloyl that was able to prevent bleeding following oral/dental surgical procedures. According to the findings, the bioadhesive could be immediately extruded into the bleeding site and shortened blood clotting time by 45%. Furthermore, it may be easily removed from the bleeding site after clotting and prevent subsequent wound harm. Guo et al. [164] prepared a hemostatic smart bioadhesive composed of hemocoagulase (the same as reptilase) and GelMA, inspired by the coagulation function of snake venom. Blood clotting time with visible light-responsive bioadhesive was about 45 s compared with 5 to 6 min without bioadhesive. Hemostatic bioadhesives achieved hemostasis in 45 s on a liver incision and 34 s on a cut rat tail, reducing blood loss by 79 and 78%, respectively.

### 4.4. Wearable Medical Devices

Nowadays, implantable and wearable medical devices such as tissue scaffolds, biodetectors, and biosensors are attracting a substantial amount of attention [178,179,180,181,182,183,184,185,186,187,188,189,190,191,192,193,194,195,196,197,198,199,200,201,202,203,204]. However, conformal and stable contact between such devices and the target tissue needs to be established. This fixation requires the use of sutures and wires, raising the risk of infection, scaffold deterioration, and subsequent injury. Smart bioadhesives have the potential to replace invasive fixing procedures with a noninvasive adhesion method. Deng et al. designed an electrical bioadhesive based on a thin layer of graphene nanocomposite that can provide rapid and on-demand detachable integration of bioelectronic devices onto a variety of wet tissues. They then successfully recorded an epicardial electrocardiogram using the synthesized bioadhesive on-site and electrically stimulated a sciatic nerve in a rat model. Zhu et al. prepared a smart ionic gelatin/PAAm/clay bioadhesive with high conductivity and high self-healing efficiency, which can be used as a capacitive pressure sensor for human motion monitoring.

## 5. Conclusions and Future Perspectives

Smart bioadhesives have become a subject of interest recently, not only because they increase the environmental sustainability and the bioadhesive’s mechanical and biological features but also the reliability of adhesion when compared with synthetic adhesives. Smart bioadhesives are stimuli-responsive materials that undergo phase and morphology variations in response to environmental stimuli (e.g., temperature, pH, electricity, light, and magnetic fields) and establish a link between therapeutic aims and drug delivery. Wound cover, tissue engineering, skin sensors, and medication delivery systems are just some applications for smart bioadhesives. Once assessing recently established smart bioadhesives, we motivated the creation, ideologies, and applications to accomplish an organized review and offer broad support for outlook materials design that show great potential in treatment areas. Though noteworthy advancement has been achieved in developing smart bioadhesives, several unsolved issues and significant obstacles in materials manufacturing and efficiency evaluation impede their useful application and industrialization. In detail: (i) the first and foremost difficulty is finding low-cost and straightforward approaches to manufacture bioinspired adhesive structures or integrating stimuli-responsive materials into adhesive; due their time-consuming nature or severe conditions, many present synthetic processes are challenging to scale up, limiting these advancements for use only in laboratories. (ii) On the other hand, one inherent problem of smart bioadhesive hydrogels is the dependence on an aqueous system, as the stimulation event is carried out by the transfer of water between the environment and the adhesive [179]. Although some studies have confirmed that a smart bioadhesive can be agitated by moisture in the air, the stimulation performance in the open air will be less than in the water environment [180]. For example, this obstacle may prevent the reconstruction of injured muscles with artificial tissue. (iii) Another challenge is the compromise between degradation and structural and functional stability of the bioadhesive after a long period for different applications. For example, we require bioadhesives that self-destruct after drug delivery to the target tissue [181]. (iv) Another difficulty that must be addressed before smart bioadhesives can be used commercially is drug delivery monitoring [182]. (v) Recently, smart bioadhesives have been utilized for several disease treatments using the transdermal drug method. However, TDD’s fundamental problems, such as the differences in drug dosing amounts between humans, parts of the skin, and gender, remain unresolved. The severe problem is that TDD does not accurately control dose absorption through injured and irritated skins, which have unpredictable drug permeability. Therefore, these groups of bioadhesives need more evaluation [183,184,185,186,187,188,189,190,191,192,193,194,195,196,197]. (vi) In contrast to various stimuli (temperature, pH, light, etc.) that have been introduced for drug delivery, a mechanical stimulus (e.g., compressive, tensile, and shear stress) can be created by the skin itself during body movement without the requirement for any external ambient stimulus. It thus makes mechanical stimulus an inexpensive drug delivery monitoring design [130]. (vii) Eventually, despite the progress in bioengineering methods for innovative bioadhesive preparation, numerous factors, such as reaction time, degradability, inflammatory, and immunological response of these materials, must be carefully evaluated to fabricate more cytocompatible bioadhesives for tissue engineering and drug delivery.

In the end, stimuli-responsive materials have broadened the scope of smart bioadhesives by boosting the accuracy in modifying therapeutic molecules’ efficacy and decreasing their off-target toxicity. Nevertheless, it is challenging to develop intelligent systems for responding to several physiological signals or external stimuli at nanoscale level. Furthermore, efforts to maintain the payload in place until desired stimulation, the ability to reach deeper layers of tissue, and minimizing unwanted tissue injury are important issues that require further progress. We hope that this study will appeal to increasing notice from research groups performing interdisciplinary research in medical science, polymer science, and engineering and which more collaborative endeavors will be devoted to the progress of intelligent bioadhesives.

## Figures and Tables

**Table 1:** Different types of light-responsive bioadhesives and their applications.

Compounds	Stimulus-Response Agents	Application	Summary	Role of Stimuli	Ref.
**Spiropyran, multishellupconversion nanoparticles**	Multishell Upconversion nanoparticles	–	The interactions between spiropyran and cell surface protein fibronectin were switchable even after 10 cycles.	By simply decreasing/increasing the excitation power density of the same 980 nm laser, cell adhesion/detachment can be switched quickly.	[51]
**Catechol** **functionalized chitosan**	MnO_2_ nanosheets	–	BMH hydrogel successfully eliminated cancer cells in vitro giant solid tumors in vivo and had effective antibacterial properties without antibiotics.	By NIR irradiation, BMH hydrogel reduced the hypoxic tumor microenvironment by degrading internal hydrogen peroxide into oxygen and simultaneously releasing the anticancer doxorubicin hydrochloride.	[53]
**Chitosan–polyvinyl** **alcohol-loaded tannic acid-TiO_2_**	–	Artificial electronic skin	Irradiation causes a change in surface wettability from hydrophobic to hydrophilic, leading to increases in electrical characteristics, mechanical strength, and adhesive properties.	Controllable swelling ratio upon irradiation with UV and visible light.	[54]
**Thiol–PEG/** **maleimide**	Upconverting nanoparticles	Tissue engineering	Preparing light-sensitive adhesive hydrogels with spatiotemporally regulated biological functions for cell culture without causing significant photodamage to the cells	Photochemical processes are activated by converting NIR light (974 nm) into local UV emission.	[55]
**PNIPAM/graphene oxide (GO)**	Graphene (808 nm)	Cell capture	The bioadhesives efficiently captured cells via the adhesive oligopeptide and released a NIR light stimulus, suitable for cell preservation and therapeutic cell delivery.	NIR light efficiently triggered cell release; continuous NIR irradiation efficiently released the cells from adhesive hydrogel.	[56]
**Dodecyl,** **chitosan**	WS_2_ nanosheets	Wound healing	Bioadhesive hydrogels with a positive charge, macropores, and alkyl chains could catch and limit microorganisms.	WS_2_ nanosheets produced heat when exposed to NIR, and the antibiotic was triggered to release at the wound site.	[57]
**PDA and PNIPAM**	PDA	Wound healing	The coating of PDA–NPs onto hydrogel surfaces was effective in cell affinity, tissue adhesiveness, and growth factor/protein immobilization ability.	Pulsatile release of drugs and quick healing (1 min) after unfavorable damage with the assistance of NIR laserirradiation.	[58]

**Table 2:** Different types of thermo-responsive bioadhesives and their applications.

Compounds	Stimulus-Response Agents	Application	Summary	Role of Stimuli	Ref.
**Pluronic^®^ 127 hydroxypropyl methylcellulose (HPMC)**	Pluronic^®^ F127	Wound infections	Ex vivo and in vivo studies showed bioadhesives with suitable antibacterial therapy of burn wound infections and anti-inflammatory activities. HPMC adhesive increased gel and bioadhesive strength	Formation of a stiff gel by increasing temperature from 4 to 32–37 °C.	[65]
**Poly(acrylic acid)(** **PAA)/PNIPAM-co-dopamine methacrylamide (PDA)**	PNIPAM	Epidermal sensors	The hydrogel with adhesive strength and self-healing ability demonstrated unusual fatigue and crack resistance properties.	Temperature-sensitive hydrogels, the lowest adhesion strength of hydrogel was at 25 °C.	[66]
**Gelatin and chondroitin sulfate**	Chondroitin sulfate	Surgical adhesive for sealing	In vivo and ex vivo, the injectable self-healing bioadhesive is used as a multifunctional tissue adhesive/sealant for closing bleeding wounds.	Exceptional tissue adherence at 37 °C diminished at low temperatures (20 °C), allowing it to detach from tissue easily.	[67]
**Polydopamine-coated Tetronics–tyramine**	Tetronic, tyramine (37 to 4 °C)	Tissue engineering	Adhesive hydrogels promoted human dermal fibroblast attachment, controlled by serum protein adsorption, creating a cell sheet after growth.	Cell sheet translocation process by changing temperature from 37 °C to 4 °C.	[68]
**Hyaluronic acid (HA), methylcellulose, polyethylene glycol (PEG)**	Methylcellulose	Surgical adhesive for sealing	Free-flowing, injectable at ambient temperature, gelation point about 40 ± 2 s, and lack of cellular toxicity	The transition of bioadhesive from sol at four °C to gel state at 37 °C.	[69]
**Catechol modified quaternized chitosan, poly(d,l-lactide)-poly(ethylene glycol)-poly(d,l-lactide) (PLEL)**	PLEL	Wound healing	The injectable thermo-sensitive adhesive hydrogel offered excellent properties as a wound dressing for promoting wound healing (only in 7 days), biocompatibility, and bioactivity through in vivo degradation, stimulated endothelial cells migration, and angiogenesis.	The temperature-triggered reversible sol (25 °C)–gel (37 °C) transition of PLEL solution.	[70]
**Galactose modified** **xyloglucan (mXG) and hydroxybutyl chitosan**	Galactose modified xyloglucan	Wound healing	According to in vivo findings, bioadhesive was an excellent anti-adhesion system for avoiding repeated adhesion following adhesiolysis, promoting wound healing and reducing scar formation.	Gelation temperature and time depended on the total solid content of bioadhesive hydrogels.	[71]
**PIPAAm, butyl methacrylate (BMA)**	PNIPAAm	Regenerative medicine and tissue engineering	Increasing BMA concentration improved the cell adhesion, owing to increased cellular protein adsorption.	Celladhesion and detachment from hydrophobized thermos-responsive brushes.	[72]
**PNIPAAm-g-chitosan**	PNIPAAm	Tissue engineering	Hydrogels showed outstanding biocompatibility to MSCs, fibroblasts, and osteoblasts, allowing cell encapsulation without toxicity.	LCST at around 30.71–32.02 °C indicated hydrogels had potential for in situ injection.	[73]
**Pluronics, hyaluronic acid, corn silk extract, and** **nanosilver**	Pluronics	Wound healing	From a biological point of view, hydrogels had good biocompatibility and exhibited antibacterial activity toward gram-positive and gram-negative bacteria.	Viscoelastic parameters changed in the temperature ranging from 25 to 40 °C.	[74]
**Collagen, chitosan, and bioactive glass**	Chitosan	Bone tissue engineering	The addition of collagen to the system resulted in larger pore size and enough interconnectivity, making it suitable for use as biomaterials for bone tissue engineering.	Gelation temperature at 37 °C.	[75]

**Table 3:** Different types of pH-responsive bioadhesives and their applications.

Compounds	Stimulus-Response Agents	Application	Summary	Role of Stimuli	Ref.
**PAA/Zinc (II) ion**	PAA and dopamine	-	Coacervate bioadhesive with good mechanical and self-healing properties.	Oxidation of catechol groups at basic pH favored the formation of strong adhesion.	[95]
**Carbon nanotubes and GO/tectomers**	Tectomer	Tissue engineering	The hybrid materials can be used as pH-switchable bioadhesive coatings and scaffolds for tumor models in ex vivo studying.	Controlled release from a pH-dependent peptidic coating.	[96]
**Chitosan-grafted-dihydrocaffeicacid/oxidized pullulan**	Chitosan-g-dihydrocaffeic acid	Drug delivery	Good injectability, a decent gelation duration, and pH-dependent equilibrated swelling ratios, morphologies, and rheological properties were observed by bioadhesive hydrogels.	At acidic conditions, the hydrogels had a larger swelling ratio and pore size than at pH 7.4.	[97]
**D-α-tocopheryl PEG 1000 succinate conjugated chitosan.**	Chitosan	Drug delivery	Invivo pharmacokinetic results demonstrated the relative bioavailability of bioadhesive micelles was effective beneficial for brain cancer therapies with the prolonged release.	A pH decrease triggered the drug release.	[98]
**Dopamine-conjugated HA/mesoporous silica**	Dopamine	Drug Delivery	In vivo studies confirmed the injection of bioadhesives could achieve high therapeutic efficiency against tumor growth while avoiding significant damage to healthy organs.	The faster release rate of the drug at pH 5.0 than at pH 7.4.	[99]
**Collagen and PEG**	Collagen	Diabetic wound repair	Bioadhesive loaded stem cell factor as an anti-inflammatory and biocompatibility dressing was used for tissue regeneration.	Effective in drug release rate.	[100]
**Chitosan and pectin**	Chitosan	Drug delivery and tissue regeneration	Based on ex vivo testing, membranes loaded with antimicrobial peptides had simultaneous antibacterial effectiveness against oral streptococci as well as cytocompatibility with both soft and hard tissue.	Temporary preventive and therapeutic distribution in the oral cavity with a ‘supply on demand’ release behavior in a pH-controlled manner	[101]
**PAA and PAAm**	PAA and PAAm	Drug delivery	In vitro findings showed dual pH-responsive bioadhesive hydrogel can release lipophilic or hydrophilic pharmaceuticals based on the pH of the environment while preventing drug metabolism, degradation, and excretion.	In alkaline or acid conditions, the bioadhesive can conduct programmable and bidirectional bending by shrinking anionic and cationic networks and asymmetric swelling.	[102]
**PAA**	PAA	Sensor	Bacterial detachment is caused by increasing brush thickness, disparity, and solution pH.	Tuning the attachment and detachment of bacteria in various pH values.	[103]

**Table 4:** Different types of electromagnetic-responsive bioadhesives and their applications.

Compounds	Stimulus-Response Agents	Application	Summary	Role of Stimuli	Ref.
**Poly [anilineTetramermethacrylamide]-co-[dopamine methacrylamide]-co-[poly(ethylene glycol) methyl ether methacrylate]}**	-	Bone tissue engineering	A conductive bioadhesive with biocompatibility and strong adhesion was prepared for regeneration of comminuted bone fracture; the adhesive strength of hydrogel was less than that of the cortical bone and showed in in vivo cytotoxicity.	Electrical conductivity of bioadhesive enhanced with the increase of AT, which improved cellular activities.	[112]
**AA and PEG dimethacrylate/GO/gelatin**	Graphene oxide	Wound healing	Adhesive hydrogel with good thermal and mechanical stability indicated viability of more than 94% for human fibroblasts, while curcumin-loaded samples showed a reduction of bacteria of 90%.	At 0 and V, the slow and fast release was achieved, while intermediate kinetics was found at 12 and V.	[113]
**Xanthan gum, chitosan, and iron oxide magnetic**	-	Muscle, skin, cartilage, and connective tissue engineering	In vitro studies showed that bioadhesive hydrogels improved fibroblasts’ growth and adherence in an external magnetic field compared to the pristine hydrogel.	In a magnetic field, adhesion and proliferation of fibroblasts were enhanced in hydrogels containing magnetic nanoparticles.	[114]
**PAA grafted gum ghatti (GGH)**	Gum ghatti	Drugs delivery by the skin	A histopathology examination demonstrated reversible changes in skin structure.	The release was observed over a two-fold increase in the drug after applying an electric stimulus.	[115]
**Nanoclay (laponite), multiwalled carbon nanotubes (CNTs), and NIPAM**	CNTs	Human motion sensing	Multifunctional conductive flexible hydrogels with self-healing, sticky, and 3D printable properties without any toxicity for the L929 cells.	Conductive bioadhesive hydrogels for wearable electronic devices revealed good electrical stability and multifunctional stretchability.	[116]
**Chitosan-aniline** **oligomer/polyvinyl alcohol**	Polyaniline	Tissue engineering	Biocompatibility testing demonstrated the conductive substrate offered the platform with more cellular activity than non-conductive materials.	Rising in drug release after electrical stimulation in comparison with non-stimulated webs.	[117]
**GO-PAA**	Graphene oxide	Artificial muscle and tissue engineering scaffold	Bioadhesive hydrogel showed good compatibility with bone marrow-derived mesenchymal stem cells.	Under the circumstance of electrical stimulation, the morphology of adherent cells was changed, and the differentiation of neural stem cells was promoted.	[118]

**Table 5:** Different types of electro-responsive bioadhesives and their applications.

Compounds	Stimulus-Response Agents	Application	Summary	Role of Stimuli	Ref.
**Thioglycolic acid, chitosan, gold nanoparticle**	Thioglycolic acid	-	Ultra-low concentrations of thrombin, as well as low molecular weight anatoxin, are detected selectively and reproducibly.	Detect early biomarkers in complex body fluid.	[122]
**Phenylboronic acid and cis -diol modified PEG**	Modified PEG	Drug delivery	The injectable, self-healing and adhesive hydrogel could have applications in 3D cell culture substrates for tissue engineering and controlled macromolecule release.	Size-dependent controlled release of proteins encapsulated within the network and the glucose-responsive release of larger proteins.	[123]
**Hyaluronic acid cross-linked with divinyl sulfone.**	Hyaluronic acid	Diabetic patients	The released insulin from glucose-responsive nanocarriers displayed a practical hypoglycemic effect for a longer time after oral administration to diabetic rats than insulin-loaded nanocarriers.	Regulation of insulin.	[124]
**2-nitroimidazole–l-cysteine–alginate**	2-nitroimidazole	Diabetic patients	Invivo experiments on type I diabetic rats showed that the hyperglycemia risk was reduced following oral administration, and a standard glucose range was maintained for a long time.	Blood glucose regulation via glucose catalysis by glucose-responsive adhesives.	[120]

**Table 6:** Different types of multi-responsive bioadhesives and their applications.

Compounds	Stimulus/Stimulus-Response Agents	Application	Summary	Role of Stimuli	Ref.
**PEG, PSMEU**	pH and thermal/PSMEU	Wound healing	Bioadhesive hydrogels were used in vivo to seal cutaneous wounds, absorb wound exudates, and promote tissue regeneration in the injured area.	Free-flowing PEG–PSMEU copolymer sols (pH 8.5, 23 °C) were converted into stable gels in the body (pH 7.4, 37 °C).	[128]
**Alginate–boronic acid conjugate**	pH- and glucose/boronic acid-diol complexation	Drug delivery systems	Alginate-BA hydrogels showed great promise in various applications, including pressure-sensitive biological glues to biomedical substrates requiring stretchability, self-healing, and multiresponsiveness.	Effect on the viscoelastic and mechanical properties of bioadhesive hydrogels.	[129]
**Dopamine functionalized 4-armed PEG (4-arm-PEG-DA) and phenylboronic acid**	pH, glucose, and dopamine triple-responsive/Dopamine and modified PEG	Drug delivery, Tissue engineering	Bioadhesive showed good adherence to tissues, and in vitro cytotoxicity experiments showed hydrogels were very cytocompatible.	The disintegration rate of hydrogel increased by decreasing pH value from 9 to 3.	[132]
**PNIPAM/PDA/clay**	Light-and thermos/PDA, PNIPAM	Electronic skin	In vitro cytotoxicity results indicated that hydrogel with high adhesiveness and biocompatibility suggested good cell affinity and biocompatibility.	Locally controllable deformation of the hydrogel by remote NIR irradiation.	[133]
**Thiolated** **chitosan and thiolated chondroitin sulfate**	pH and redox/Amino groups, carboxyl and sulfate groups	Wound healing and tissue engineering	Multilayer systems with disulfide bonds aided tuning cell contact, film degradation, and controlled release of bioactive compounds.	Cross-linking in alkaline pH or reduction of disulfide bonds changed mechanical and surface properties and cell function.	[134]
**Collagen (COL), guar gum (GG), PNIPAM, GO**	Light and thermal/PNIPAM and GO	Wound healing, wearable electronic devices, and sensors.	A bioadhesive hydrogel with many functions was synthesized, including quick wound healing, super-ductility, injectability, remoldability, conductive, thermo-sensitive, NIR-responsive, and accelerated wound healing.	Phase change occurs shortly after touches the human body.	[135]
**PAA, oligo(ethylene** **glycol) methacrylate, 2-(2-methoxyethoxy) ethyl methacrylate, chitosan**	pH and thermal/PAA (pH-sensitive) and oligo(ethylene glycol) methacrylate and 2-(2-methoxyethoxy) ethyl methacrylate (Thermal sensitive)	Drug delivery	In vitro cytotoxicity studies confirmed that hydrogels had excellent cell compatibility, with 5-Fu-loaded hydrogels having a lower cell growth inhibition efficiency for normal LO2 cells but a higher cell growth inhibition efficiency for cancer HepG2 cells than pure 5-Fu at the same drug concentration.	The value of medication released was low in an acidic environment (pH 1.2) but high in a neutral environment.	[136]
**Poly (1-butyl-3-vinylimidazolium bis(trifluoromethanesulfonyl)imide) ([PBVIm] [TFSI])**	Strain and electric	Utilized in clothing to monitor various body movements	Membranes possessed washable, comfortable, good mechanical properties and satisfactory moisture proof sensing performance.	-	[137]
**1-vinyl-3-butylimidazolium bromide ([VBIM+] Br-) ionic liquid, vinyl-modified lignin (v-lignin), acrylamide (AM), borax, ammonium persulfate**	Strain and thermoresponsive	Electronic skin, human–machine interface, and remote medical healthcare	Hydrogel showed high stretchability, excellent toughness, and impressive stress loading-unloading cyclic stability.	Motion capture and gesture identification by the hydrogel strain sensor.	[138]
**Lignin/poly(ionic liquids)/** **3-butyl-1-isopropyl-1H-imidazol-3-ium bromide/1-vinylimidazole and bromobutane**	pH and temperature responsive	Drug delivery	The hybrid hydrogel was more successful at killing malignant cells in an invitro cytotoxicity and drug release testing.	Drug release occurred at intracellular acidic pH.	[139]
**1-vinyl-3-butylimidazolium tetrafluoroborate/1-butyl-3-methylimidazolium tetrafluoroborate**	Strain and light	Reusable wearable electronics	Ionogel integrated excellent mechanical properties, ultra-strong adhesive, self- healing ability, and recyclability.	Detection of physical motion and physiological signals of human body.	[140]
**Cetylpyridinium salicylate/cetpylpyridinium chloride**	pH and temperature	Drug delivery	The preparation of hybrid pharmaceutical ionogels through encapsulation of the chemotherapeutic drug imatinib mesylate within the ionogel matrix.	The maximum release drug was conducted at an acidic pH at 37 °C.	[141]
**Dual-cross-linked ionohydrogel**	Temperature and strain	Wearable ionotronic devices	The bioadhesives possessed excellent mechanical properties, transparency, high ionic conductivity, and robust adhesion, along with the advantages of superior antifreezing and long-term antidehydration properties.		[142]
**1-methyl-3-(oxiran-2-ylmethyl)-1H-imidazol-3-ium chloride/methoxy polyethylenglycol-aldehyde/chitosan**	Magnetic, pH responsive	Drug delivery	The findings of the cytotoxicity assay demonstrated that medications loaded nanocarriers have a higher cytotoxicity effect than free drugs.	pH-responsive branched nanocarrier for co-delivery of DOX and MTX.	[143]

**Table 7:** Clinical applications of smart bioadhesives.

Compounds	Stimuli	Application	Summary	Ref
**Poly(glycerol sebacate)-co-poly(ethylene glycol)-g-catechol**	Photothermal	Wound closure	Bioadhesives perform superior wound closure and healing of skin incisions than medical glue and surgical suture, with good hemostasis and a high killing ratio of bacteria.	[157]
**Ferric ion, protocatechualdehyde containing catechol and aldehyde groups and quaternized chitosan**	NIR responsiveness	Wound closure	Bioadhesives presents good biocompatibility, hemostasis, antibacterial activity, injectability, and multifunctional adhesiveness.	[158]
**Hyaluronic acid-graft-dopamine andreduced graphene oxide**	NIR responsiveness	Drug delivery	Bioadhesive hemostatic antioxidative conductive hydrogels with sustained drug release properties are an ideal wound dressing for promoting full-thickness skin regeneration.	[159]
**Poly(*N*-isopropylacrylamide) terminated with catechols/** **polypyrrole nanoparticles**	pH, temperature, and NIR light–responsive	Drug delivery	Bioadhesive with multi-responsive behavior, especially NIR light response, can be profitable in removable sealant materials and remotely controlled release systems.	[160]
**Graphene aerogel/poly(*N*-isopropylacrylamide)hydrogel/polydopamine nanoparticles**	Thermo- and NIR responsiveness	Drug delivery	Correlation between the drug release and the resistance allowed the drug-release behavior of the bioadhesive hydrogels to be monitored using electrical signals	[161]
**Alginate/PNIPAm**	Thermoresponsive	Sealing leakage and wound healing	Inspired by embryonic wound contraction, bioadhesive can support skin wound healing with stretchability, toughness, tissue adhesion, and antimicrobial function.	[162]
**Gelatin Methacryloyl (GelMA)/PhenylIsothiocyanate-Modified Gelatin**	Light-responsive	Hemostasis	The produced bioadhesive with injectability and immediate hemostatic effect can be used as a fast cross-linkable hemostatic agent for irregular wounds in oral/dental surgical procedures.	[163]
**Hemocoagulase/GelMA**	Visible light-responsive	Hemostasis	The bioadhesives resulted in fast hemostasis and tissue sealing through the activation and aggregation of platelets as well as the effective transformation of fibrinogen into fibrin.	[164]
**GO/poly(vinylalcohol)** **/** **PAA grafted with *N*-hydroxysuccinimide ester**	Electro-responsive	Bioelectronic	The obtained bioadhesive with biocompatibility, applicability, mechanical and electrical stability, and recording and stimulation functionalities can be used to improve tissue–device integration and enhance the performance of biointegrated electronic devices.	[155]
**Gelatin/PAAm/Clay hydrogel**	Salt ions, pH, and stress	BioSensor	A capacitive pressure sensor with ability of high conductivity, high self-healing efficiency, and robust adhesion has been designed for monitoring human motions.	[165]

## References

[1] Li X., Su X. (2018). Multifunctional smart hydrogels: Potential in tissue engineering and cancer therapy. J. Mater. Chem. B.

[2] Khanlari S., Dubé M.A. (2013). Bioadhesives: A review. Macromol. React. Eng..

[3] Ramesh M., Kumar L.R. (2020). Bioadhesives, Green Adhesives: Preparation, Properties and Applications.

[4] Chopra H., Kumar S., Singh I. (2020). Bioadhesive Hydrogels and Their Applications. Bioadhesives in Drug Delivery.

[5] Pinnaratip R., Bhuiyan M.S.A., Meyers K., Rajachar R.M., Lee B.P. (2019). Multifunctional biomedical adhesives. Adv. Healthc. Mater..

[6] Tavakoli S., Mokhtari H., Kharaziha M., Kermanpur A., Talebi A., Moshtaghian J. (2020). A multifunctional nanocomposite spray dressing of Kappa-carrageenan-polydopamine modified ZnO/L-glutamic acid for diabetic wounds. Mater. Sci. Eng. C.

[7] Rajabi N., Kharaziha M., Emadi R., Zarrabi A., Mokhtari H., Salehi S. (2020). An adhesive and injectable nanocomposite hydrogel of thiolated gelatin/gelatin methacrylate/Laponite^®^ as a potential surgical sealant. J. Colloid Interface Sci..

[8] Cabane E., Zhang X., Langowska K., Palivan C.G., Meier W. (2012). Stimuli-responsive polymers and their applications in nanomedicine. Biointerphases.

[9] El-Sherbiny I.M., Khalil I.A., Ali I.H. (2018). Updates on stimuli-responsive polymers: Synthesis approaches and features. Polymer Gels.

[10] Bruschi M.L., Borghi-Pangoni F.B., Junqueira M.V., de Souza Ferreira S.B. (2017). Nanostructured therapeutic systems with bioadhesive and thermoresponsive properties. Nanostructures for Novel Therapy.

[11] Dey A., Bhattacharya P., Neogi S. (2020). Bioadhesives in Biomedical Applications: A Critical Review. Rev. Adhes. Adhes..

[12] Duan W., Bian X., Bu Y. (2021). Applications of bioadhesives: A mini review. Front. Bioeng. Biotechnol..

[13] Park J., Kim Y., Chun B., Seo J. (2021). Rational engineering and applications of functional bioadhesives in biomedical engineering. Biotechnol. J..

[14] Shi Q., Liu H., Tang D., Li Y., Li X., Xu F. (2019). Bioactuators based on stimulus-responsive hydrogels and their emerging biomedical applications. NPG Asia Mater..

[15] Bratek-Skicki A. (2021). Towards a new class of stimuli-responsive polymer-based materials–Recent advances and challenges. Appl. Surf. Sci. Adv..

[16] Beaussart A., Ngo T.C., Derclaye S., Kalinova R., Mincheva R., Dubois P., Leclère P., Dufrêne Y.F. (2014). Chemical force microscopy of stimuli-responsive adhesive copolymers. Nanoscale.

[17] Sharifzadeh G., Hosseinkhani H. (2017). Biomolecule-responsive hydrogels in medicine. Adv. Healthc. Mater..

[18] Aguilar M.R., San Román J. (2019). Introduction to smart polymers and their applications. Smart Polymers and Their Applications.

[19] Shoukat H., Buksh K., Noreen S., Pervaiz F., Maqbool I. (2021). Hydrogels as potential drug-delivery systems: Network design and applications. Ther. Deliv..

[20] El-Husseiny H.M., Mady E.A., Hamabe L., Abugomaa A., Shimada K., Yoshida T., Tanaka T., Yokoi A., Elbadawy M., Tanaka R. (2022). Smart/stimuli-responsive hydrogels: Cutting-edge platforms for tissue engineering and other biomedical applications. Mater. Today Bio.

[21] Pourjavadi A., Heydarpour R., Tehrani Z.M. (2021). Multi-stimuli-responsive hydrogels and their medical applications. New J. Chem..

[22] Ding M., Jing L., Yang H., Machnicki C., Fu X., Li K., Wong I., Chen P.-Y. (2020). Multifunctional soft machines based on stimuli-responsive hydrogels: From freestanding hydrogels to smart integrated systems. Mater. Today Adv..

[23] Hwang I., Kim H.N., Seong M., Lee S.H., Kang M., Yi H., Bae W.G., Kwak M.K., Jeong H.E. (2018). Multifunctional smart skin adhesive patches for advanced health care. Adv. Healthc. Mater..

[24] Guillon E., Das D., Jülich D., Hassan A.-R., Geller H., Holley S. (2020). Fibronectin is a smart adhesive that both influences and responds to the mechanics of early spinal column development. Elife.

[25] Pathak K., Malviya R. (2020). Introduction, Theories and Mechanisms of Bioadhesion. Bioadhesives in Drug Delivery.

[26] Lenaerts V.M., Gurny R. (1989). Bioadhesive Drug Delivery Systems.

[27] Kaurav H., HariKumar S., Kaur A. (2012). Mucoadhesive microspheres as carriers in drug delivery: A review. Int. J. Drug Dev. Res..

[28] Masareddy R.S., Patil A.S., Gadad A.P. (2021). Bioadhesive Nanoparticulate Drug Delivery System. Nanopharmaceutical Advanced Delivery Systems.

[29] Bouten P.J., Zonjee M., Bender J., Yauw S.T., van Goor H., van Hest J.C., Hoogenboom R. (2014). The chemistry of tissue adhesive materials. Prog. Polym. Sci..

[30] Huang S., Kong X., Xiong Y., Zhang X., Chen H., Jiang W., Niu Y., Xu W., Ren C. (2020). An overview of dynamic covalent bonds in polymer material and their applications. Eur. Polym. J..

[31] Zhang H., Zhao T., Newland B., Duffy P., Annaidh A.N., O’Cearbhaill E.D., Wang W. (2015). On-demand and negative-thermo-swelling tissue adhesive based on highly branched ambivalent PEG–catechol copolymers. J. Mater. Chem. B.

[32] Jin S.G., Kim K.S., Kim D.W., Kim D.S., Seo Y.G., Go T.G., Youn Y.S., Kim J.O., Yong C.S., Choi H.-G. (2016). Development of a novel sodium fusidate-loaded triple polymer hydrogel wound dressing: Mechanical properties and effects on wound repair. Int. J. Pharm..

[33] Korde J.M., Kandasubramanian B. (2018). Biocompatible alkyl cyanoacrylates and their derivatives as bio-adhesives. Biomater. Sci..

[34] Zhu W., Chuah Y.J., Wang D.-A. (2018). Bioadhesives for internal medical applications: A review. Acta Biomater..

[35] Schricker S.R., Palacio M.L., Bhushan B. (2012). Designing nanostructured block copolymer surfaces to control protein adhesion. Philos. Trans. Royal Soc. A.

[36] Rizwan M., Yahya R., Hassan A., Yar M., Azzahari A.D., Selvanathan V., Sonsudin F., Abouloula C.N. (2017). pH sensitive hydrogels in drug delivery: Brief history, properties, swelling, and release mechanism, material selection and applications. Polymers.

[37] Karolewicz B. (2016). A review of polymers as multifunctional excipients in drug dosage form technology. Saudi Pharm. J..

[38] Mahinroosta M., Farsangi Z.J., Allahverdi A., Shakoori Z. (2018). Hydrogels as intelligent materials: A brief review of synthesis, properties and applications. Mater. Today Chem..

[39] Ebara M., Kotsuchibashi Y., Narain R., Idota N., Kim Y.-J., Hoffman J.M., Uto K., Aoyagi T. (2014). Smart Biomaterials.

[40] Lu W., Le X., Zhang J., Huang Y., Chen T. (2017). Supramolecular shape memory hydrogels: A new bridge between stimuli-responsive polymers and supramolecular chemistry. Chem. Soc. Rev..

[41] Michal B.T., Spencer E.J., Rowan S.J. (2016). Stimuli-responsive reversible two-level adhesion from a structurally dynamic shape-memory polymer. ACS Appl. Mater. Interfaces.

[42] Zhang J., Cui Z., Field R., Moloney M.G., Rimmer S., Ye H. (2015). Thermo-responsive microcarriers based on poly (*N*-isopropylacrylamide). Eur. Polym. J..

[43] Swift T., Swanson L., Geoghegan M., Rimmer S. (2016). The pH-responsive behaviour of poly (acrylic acid) in aqueous solution is dependent on molar mass. Soft Matter.

[44] Martella D., Nocentini S., Micheletti F., Wiersma D.S., Parmeggiani C. (2019). Polarization-dependent deformation in light responsive polymers doped by dichroic dyes. Soft Matter.

[45] Sénéchal V., Saadaoui H., Rodriguez-Hernandez J., Drummond C. (2017). Electro-responsive polyelectrolyte-coated surfaces. Faraday Discuss..

[46] Hao Y., Meng J., Wang S. (2017). Photo-responsive polymer materials for biological applications. Chin. Chem. Lett..

[47] Manouras T., Vamvakaki M. (2017). Field responsive materials: Photo-, electro-, magnetic-and ultrasound-sensitive polymers. Polym. Chem..

[48] Hao Y., Cui H., Meng J., Wang S. (2018). Photo-responsive smart surfaces with controllable cell adhesion. J. Photochem. Photobiol. A Chem..

[49] He F., Yang G., Yang P., Yu Y., Lv R., Li C., Dai Y., Gai S., Lin J. (2015). A new single 808 nm NIR light-induced imaging-guided multifunctional cancer therapy platform. Adv. Funct. Mater..

[50] Kasiński A., Zielińska-Pisklak M., Oledzka E., Sobczak M. (2020). Smart hydrogels–synthetic stimuli-responsive antitumor drug release systems. Int. J. Nanomed..

[51] Li W., Chen Z., Zhou L., Li Z., Ren J., Qu X. (2015). Noninvasive and reversible cell adhesion and detachment via single-wavelength near-infrared laser mediated photoisomerization. J. Am. Chem. Soc..

[52] Bian Q., Chen S., Xing Y., Yuan D., Lv L., Wang G. (2018). Host-guest self-assembly toward reversible visible-light-responsive switching for bacterial adhesion. Acta Biomater..

[53] Wang S., Zheng H., Zhou L., Cheng F., Liu Z., Zhang H., Zhang Q. (2020). Injectable redox and light responsive MnO_2_ hybrid hydrogel for simultaneous melanoma therapy and multidrug-resistant bacteria-infected wound healing. Biomaterials.

[54] Ryplida B., Lee K.D., In I., Park S.Y. (2019). Light-Induced Swelling-Responsive Conductive, Adhesive, and Stretchable Wireless Film Hydrogel as Electronic Artificial Skin. Adv. Funct. Mater..

[55] Zheng Y., Chen Z., Jiang Q., Feng J., Wu S., Del Campo A. (2020). Near-infrared-light regulated angiogenesis in a 4D hydrogel. Nanoscale.

[56] Li W., Wang J., Ren J., Qu X. (2013). 3D graphene oxide–polymer hydrogel: Near-infrared light-triggered active scaffold for reversible cell capture and on-demand release. Adv. Mater..

[57] Yang N., Zhu M., Xu G., Liu N., Yu C. (2020). A near-infrared light-responsive multifunctional nanocomposite hydrogel for efficient and synergistic antibacterial wound therapy and healing promotion. J. Mater. Chem. B.

[58] Han L., Zhang Y., Lu X., Wang K., Wang Z., Zhang H. (2016). Polydopamine nanoparticles modulating stimuli-responsive PNIPAM hydrogels with cell/tissue adhesiveness. ACS Appl. Mater. Interfaces.

[59] Abueva C.D., Chung P.-S., Ryu H.-S., Park S.-Y., Woo S.H. (2020). Photoresponsive Hydrogels as Drug Delivery Systems. Med. Lasers Eng. Basic Res. Clin. Appl..

[60] Lv S.-W., Liu Y., Xie M., Wang J., Yan X.-W., Li Z., Dong W.-G., Huang W.-H. (2016). Near-infrared light-responsive hydrogel for specific recognition and photothermal site-release of circulating tumor cells. ACS Nano.

[61] Jiang Z., Tan M.L., Taheri M., Yan Q., Tsuzuki T., Gardiner M.G., Diggle B., Connal L.A. (2020). Strong, Self-Healable, and Recyclable Visible-Light-Responsive Hydrogel Actuators. Angew. Chem..

[62] Lv Z., He S., Wang Y., Zhu X. (2021). Noble Metal Nanomaterials for NIR-Triggered Photothermal Therapy in Cancer. Adv. Healthc. Mater..

[63] Bi J., Song K., Wu S., Zhang Y., Wang Y., Liu T. (2019). Effect of thermal-responsive surfaces based on PNIPAAm on cell adsorption/desorption. Int. J. Polym. Mater. Polym. Biomater..

[64] Abuwatfa W.H., Awad N.S., Pitt W.G., Husseini G.A. (2022). Thermosensitive Polymers and Thermo-Responsive Liposomal Drug Delivery Systems. Polymers.

[65] Arafa M.G., El-Kased R.F., Elmazar M. (2018). Thermoresponsive gels containing gold nanoparticles as smart antibacterial and wound healing agents. Sci. Rep..

[66] Zhang R., Ruan H., Fu Q., Zhu X., Yao Y. (2020). A high strain, adhesive, self-healable poly (acrylic acid) hydrogel with temperature sensitivity as an epidermal sensor. Mater. Adv..

[67] Zhou L., Dai C., Fan L., Jiang Y., Liu C., Zhou Z., Guan P., Tian Y., Xing J., Li X. (2021). Injectable Self-Healing Natural Biopolymer-Based Hydrogel Adhesive with Thermoresponsive Reversible Adhesion for Minimally Invasive Surgery. Adv. Funct. Mater..

[68] Lee Y.B., Shin Y.M., Kim E.M., Lee J.-Y., Lim J., Kwon S.K., Shin H. (2016). Mussel adhesive protein inspired coatings on temperature-responsive hydrogels for cell sheet engineering. J. Mater. Chem. B.

[69] Sultana T., Gwon J.-G., Lee B.-T. (2020). Thermal stimuli-responsive hyaluronic acid loaded cellulose based physical hydrogel for post-surgical de novo peritoneal adhesion prevention. Mater. Sci. Eng. C.

[70] Zheng Z., Bian S., Li Z., Zhang Z., Liu Y., Zhai X., Pan H., Zhao X. (2020). Catechol modified quaternized chitosan enhanced wet adhesive and antibacterial properties of injectable thermo-sensitive hydrogel for wound healing. Carbohydr. Polym..

[71] Zhang E., Guo Q., Ji F., Tian X., Cui J., Song Y., Sun H., Li J., Yao F. (2018). Thermoresponsive polysaccharide-based composite hydrogel with antibacterial and healing-promoting activities for preventing recurrent adhesion after adhesiolysis. Acta Biomater..

[72] Nagase K., Hatakeyama Y., Shimizu T., Matsuura K., Yamato M., Takeda N., Okano T. (2013). Hydrophobized thermoresponsive copolymer brushes for cell separation by multistep temperature change. Biomacromolecules.

[73] Wu S.-W., Liu X., Miller A.L., Cheng Y.-S., Yeh M.-L., Lu L. (2018). Strengthening injectable thermo-sensitive NIPAAm-g-chitosan hydrogels using chemical cross-linking of disulfide bonds as scaffolds for tissue engineering. Carbohydr. Polym..

[74] Makvandi P., Ali G.W., Della Sala F., Abdel-Fattah W.I., Borzacchiello A. (2019). Biosynthesis and characterization of antibacterial thermosensitive hydrogels based on corn silk extract, hyaluronic acid and nanosilver for potential wound healing. Carbohydr. Polym..

[75] Moreira C.D., Carvalho S.M., Mansur H.S., Pereira M.M. (2016). Thermogelling chitosan–collagen–bioactive glass nanoparticle hybrids as potential injectable systems for tissue engineering. Mater. Sci. Eng. C.

[76] Umapathi R., Reddy P.M., Rani A., Venkatesu P. (2018). Influence of additives on thermoresponsive polymers in aqueous media: A case study of poly (*N*-isopropylacrylamide). Phys. Chem. Chem. Phys..

[77] Kim Y.-J., Matsunaga Y.T. (2017). Thermo-responsive polymers and their application as smart biomaterials. J. Mater. Chem. B.

[78] Chen Y., Cheng W., Teng L., Jin M., Lu B., Ren L., Wang Y. (2018). Graphene oxide hybrid supramolecular hydrogels with self-healable, bioadhesive and stimuli-responsive properties and drug delivery application. Macromol. Mater. Eng..

[79] Mantha S., Pillai S., Khayambashi P., Upadhyay A., Zhang Y., Tao O., Pham H.M., Tran S.D. (2019). Smart hydrogels in tissue engineering and regenerative medicine. Materials.

[80] Xue X., Thiagarajan L., Braim S., Saunders B.R., Shakesheff K.M., Alexander C. (2017). Upper critical solution temperature thermo-responsive polymer brushes and a mechanism for controlled cell attachment. J. Mater. Chem. B.

[81] Nagase K., Uchikawa N., Hirotani T., Akimoto A.M., Kanazawa H. (2020). Thermoresponsive anionic copolymer brush-grafted surfaces for cell separation. Colloids Surf. B Biointerfaces.

[82] Shen Y., Li G., Ma Y., Yu D., Sun J., Li Z. (2015). Smart surfaces based on thermo-responsive polymer brushes prepared from L-alanine derivatives for cell capture and release. Soft Matter.

[83] Saravanan S., Vimalraj S., Anuradha D. (2018). Chitosan based thermoresponsive hydrogel containing graphene oxide for bone tissue repair. Biomed. Pharmacother..

[84] Wang Q., Feng Y., He M., Huang Y., Zhao W., Zhao C. (2018). Thermoresponsive Antibacterial Surfaces Switching from Bacterial Adhesion to Bacterial Repulsion. Macromol. Mater. Eng..

[85] Sutton A., Shirman T., Timonen J.V., England G.T., Kim P., Kolle M., Ferrante T., Zarzar L.D., Strong E., Aizenberg J. (2017). Photothermally triggered actuation of hybrid materials as a new platform for In Vitro cell manipulation. Nat. Commun..

[86] Sun X., Tyagi P., Agate S., McCord M.G., Lucia L.A., Pal L. (2020). Highly tunable bioadhesion and optics of 3D printable PNIPAm/cellulose nanofibrils hydrogels. Carbohydr. Polym..

[87] Ferber S., Behrens A.M., McHugh K.J., Rosenberg E.M., Linehan A.R., Sugarman J.L., Jayawardena H.S.N., Langer R., Jaklenec A. (2018). Evaporative cooling hydrogel packaging for storing biologics outside of the cold chain. Adv. Healthc. Mater..

[88] Xu X., Liu Y., Fu W., Yao M., Ding Z., Xuan J., Li D., Wang S., Xia Y., Cao M. (2020). Poly (*N*-isopropylacrylamide)-based thermoresponsive composite hydrogels for biomedical applications. Polymers.

[89] Luckanagul J.A., Pitakchatwong C., Bhuket P.R.N., Muangnoi C., Rojsitthisak P., Chirachanchai S., Wang Q., Rojsitthisak P. (2018). Chitosan-based polymer hybrids for thermo-responsive nanogel delivery of curcumin. Carbohydr. Polym..

[90] Kumar T.M., Paul W., Sharma C.P., Kuriachan M. (2005). Bioadhesive, pH responsive micromatrix for oral delivery of insulin. Trends Biomater. Artif. Organs.

[91] Dai S., Ravi P., Tam K.C. (2008). pH-Responsive polymers: Synthesis, properties and applications. Soft Matter.

[92] Yang J., Dahlström C., Edlund H., Lindman B., Norgren M. (2019). pH-responsive cellulose—Chitosan nanocomposite films with slow release of chitosan. Cellulose.

[93] Desbrières J., Guibal E. (2018). Chitosan for wastewater treatment. Polym. Int..

[94] Ofridam F., Tarhini M., Lebaz N., Gagniere E., Mangin D., Elaïssari A. (2021). pH-sensitive polymers: Classification and some fine potential applications. Polym. Adv. Technol..

[95] Wang W., Xu Y., Li A., Li T., Liu M., von Klitzing R., Ober C.K., Kayitmazer A.B., Li L., Guo X. (2015). Zinc induced polyelectrolyte coacervate bioadhesive and its transition to a self-healing hydrogel. RSC Adv..

[96] Garriga R., Jurewicz I., Seyedin S., Bardi N., Totti S., Matta-Domjan B., Velliou E.G., Alkhorayef M.A., Cebolla V.L., Razal J.M. (2017). Multifunctional, biocompatible and pH-responsive carbon nanotube-and graphene oxide/tectomer hybrid composites and coatings. Nanoscale.

[97] Liang Y., Zhao X., Ma P.X., Guo B., Du Y., Han X. (2019). pH-responsive injectable hydrogels with mucosal adhesiveness based on chitosan-grafted-dihydrocaffeic acid and oxidized pullulan for localized drug delivery. J. Colloid Interface Sci..

[98] Agrawal P., Singh R.P., Sharma G., Mehata A.K., Singh S., Rajesh C.V., Pandey B.L., Koch B., Muthu M.S. (2017). Bioadhesive micelles of d-α-tocopherol polyethylene glycol succinate 1000: Synergism of chitosan and transferrin in targeted drug delivery. Colloids Surf. B Biointerfaces.

[99] Wu D., Shi X., Zhao F., Chilengue S.T.F., Deng L., Dong A., Kong D., Wang W., Zhang J. (2019). An injectable and tumor-specific responsive hydrogel with tissue-adhesive and nanomedicine-releasing abilities for precise locoregional chemotherapy. Acta Biomater..

[100] Zhang L., Zhou Y., Su D., Wu S., Zhou J., Chen J. (2021). Injectable, Self-healing and pH Responsive Stem Cell Factor Loaded Collagen Hydrogel as Dynamic Bioadhesive Dressing for Diabetic Wound Repair. J. Mater. Chem. B.

[101] Boda S.K., Fischer N.G., Ye Z., Aparicio C. (2020). Dual Oral Tissue Adhesive Nanofiber Membranes for pH-Responsive Delivery of Antimicrobial Peptides. Biomacromolecules.

[102] Han Z., Wang P., Mao G., Yin T., Zhong D., Yiming B., Hu X., Jia Z., Nian G., Qu S. (2020). Dual pH-responsive hydrogel actuator for lipophilic drug delivery. ACS Appl. Mater. Interfaces.

[103] Yadav V., Jaimes-Lizcano Y.A., Dewangan N.K., Park N., Li T.-H., Robertson M.L., Conrad J.C. (2017). Tuning bacterial attachment and detachment via the thickness and dispersity of a pH-responsive polymer brush. ACS Appl. Mater. Interfaces.

[104] He J., Zhang Z., Yang Y., Ren F., Li J., Zhu S., Ma F., Wu R., Lv Y., He G. (2021). Injectable Self-Healing Adhesive pH-Responsive Hydrogels Accelerate Gastric Hemostasis and Wound Healing. Nano-Micro Lett..

[105] Baloğlu E., Özyazıcı M., Hızarcıoğlu S.Y., Karavana H.A. (2003). An in vitro investigation for vaginal bioadhesive formulations: Bioadhesive properties and swelling states of polymer mixtures. Il Farmaco.

[106] Pan G., Li F., He S., Li W., Wu Q., He J., Ruan R., Xiao Z., Zhang J., Yang H. (2022). Mussel-and Barnacle Cement Proteins-Inspired Dual-Bionic Bioadhesive with Repeatable Wet-Tissue Adhesion, Multimodal Self-Healing, and Antibacterial Capability for Nonpressing Hemostasis and Promoted Wound Healing. Adv. Funct. Mater..

[107] Xie C., Li P., Han L., Wang Z., Zhou T., Deng W., Wang K., Lu X. (2017). Electroresponsive and cell-affinitive polydopamine/polypyrrole composite microcapsules with a dual-function of on-demand drug delivery and cell stimulation for electrical therapy. NPG Asia Mater..

[108] Ali I., Xudong L., Xiaoqing C., Zhiwei J., Pervaiz M., Weimin Y., Haoyi L., Sain M. (2019). A review of electro-stimulated gels and their applications: Present state and future perspectives. Mater. Sci. Eng. C.

[109] Palza H., Zapata P.A., Angulo-Pineda C. (2019). Electroactive smart polymers for biomedical applications. Materials.

[110] Romasanta L.J., López-Manchado M.A., Verdejo R. (2015). Increasing the performance of dielectric elastomer actuators: A review from the materials perspective. Prog. Polym. Sci..

[111] Adesanya K., Vanderleyden E., Embrechts A., Glazer P., Mendes E., Dubruel P. (2014). Properties of electrically responsive hydrogels as a potential dynamic tool for biomedical applications. J. Appl. Polym. Sci..

[112] Yan H., Li L., Wang Z., Wang Y., Guo M., Shi X., Yeh J.-M., Zhang P. (2019). Mussel-inspired conducting copolymer with aniline tetramer as intelligent biological adhesive for bone tissue engineering. ACS Biomater. Sci. Eng..

[113] di Luca M., Vittorio O., Cirillo G., Curcio M., Czuban M., Farfalla A., Hampel S., Nicoletta F.P., Iemma F. (2018). Electro-responsive graphene oxide hydrogels for skin bandages: The outcome of gelatin and trypsin immobilization. Int. J. Pharm..

[114] Rao K.M., Kumar A., Han S.S. (2018). Polysaccharide-based magnetically responsive polyelectrolyte hydrogels for tissue engineering applications. J. Mater. Sci. Technol..

[115] Birajdar R.P., Patil S.B., Alange V.V., Kulkarni R.V. (2019). Electro-responsive polyacrylamide-grafted-gum ghatti copolymer for transdermal drug delivery application. J. Macromol. Sci. Part A.

[116] Deng Z., Hu T., Lei Q., He J., Ma P.X., Guo B. (2019). Stimuli-responsive conductive nanocomposite hydrogels with high stretchability, self-healing, adhesiveness, and 3D printability for human motion sensing. ACS Appl. Mater. Interfaces.

[117] Bagheri B., Zarrintaj P., Samadi A., Zarrintaj R., Ganjali M.R., Saeb M.R., Mozafari M., Park O.O., Kim Y.C. (2020). Tissue engineering with electrospun electro-responsive chitosan-aniline oligomer/polyvinyl alcohol. Int. J. Biol. Macromol..

[118] Qiao K., Guo S., Zheng Y., Xu X., Meng H., Peng J., Fang Z., Xie Y. (2018). Effects of graphene on the structure, properties, electro-response behaviors of GO/PAA composite hydrogels and influence of electro-mechanical coupling on BMSC differentiation. Mater. Sci. Eng. C.

[119] Vázquez-González M., Willner I. (2020). Stimuli-Responsive Biomolecule-Based Hydrogels and Their Applications. Angew. Chem. Int. Ed..

[120] Zhou X., Wu H., Long R., Wang S., Huang H., Xia Y., Wang P., Lei Y., Cai Y., Cai D. (2020). Oral delivery of insulin with intelligent glucose-responsive switch for blood glucose regulation. J. Nanobiotech..

[121] Turner J.G., White L.R., Estrela P., Leese H.S. (2021). Hydrogel-Forming Microneedles: Current Advancements and Future Trends. Macromol. Biosci..

[122] Lin Z.T., Gu J., Li C.H., Lee T.R., Xie L., Chen S., Cao P.Y., Jiang S., Yuan Y., Hong X. (2017). A nanoparticle-decorated biomolecule-responsive polymer enables robust signaling cascade for biosensing. Adv. Mater..

[123] Yesilyurt V., Webber M.J., Appel E.A., Godwin C., Langer R., Anderson D.G. (2016). Injectable self-healing glucose-responsive hydrogels with pH-regulated mechanical properties. Adv. Mater..

[124] Li L., Jiang G., Yu W., Liu D., Chen H., Liu Y., Huang Q., Tong Z., Yao J., Kong X. (2016). A composite hydrogel system containing glucose-responsive nanocarriers for oral delivery of insulin. Mater. Sci. Eng. C.

[125] Sigen A., Xu Q., Johnson M., Creagh-Flynn J., Venet M., Zhou D., Lara-Sáez I., Tai H., Wang W. (2021). An injectable multi-responsive hydrogel as self-healable and on-demand dissolution tissue adhesive. Appl. Mater. Today.

[126] Aminu N., Toh S. (2017). Applicability of nanoparticles-hydrogel composite in treating periodontal diseases and beyond. Asian J. Pharm. Clin. Res..

[127] Alvarez-Lorenzo C., Bromberg L., Concheiro A. (2009). Light-sensitive intelligent drug delivery systems. Photochem. Photobiol..

[128] Le T.M.D., Duong H.T.T., Thambi T., Giang Phan V., Jeong J.H., Lee D.S. (2018). Bioinspired pH-and temperature-responsive injectable adhesive hydrogels with polyplexes promotes skin wound healing. Biomacromolecules.

[129] Hong S.H., Kim S., Park J.P., Shin M., Kim K., Ryu J.H., Lee H. (2018). Dynamic bonds between boronic acid and alginate: Hydrogels with stretchable, self-healing, stimuli-responsive, remoldable, and adhesive properties. Biomacromolecules.

[130] Abebe M.W., Appiah-Ntiamoah R., Kim H. (2020). Gallic acid modified alginate self-adhesive hydrogel for strain responsive transdermal delivery. Int. J. Biol. Macromol..

[131] Li S., Xu J., Yao G., Liu H. (2019). Self-adhesive, self-healable, and triple-responsive hydrogel doped with polydopamine as an adsorbent toward methylene blue. Ind. Eng. Chem. Res..

[132] Shan M., Gong C., Li B., Wu G. (2017). A pH, glucose, and dopamine triple-responsive, self-healable adhesive hydrogel formed by phenylborate–catechol complexation. Polym. Chem..

[133] Di X., Kang Y., Li F., Yao R., Chen Q., Hang C., Xu Y., Wang Y., Sun P., Wu G. (2019). Poly (*N*-isopropylacrylamide)/ polydopamine/clay nanocomposite hydrogels with stretchability, conductivity, and dual light-and thermo-responsive bending and adhesive properties. Colloids Surf. B Biointerfaces.

[134] Esmaeilzadeh P., Köwitsch A., Liedmann A., Menzel M., Fuhrmann B., Schmidt G., Klehm J., Groth T. (2018). Stimuli-responsive multilayers based on thiolated polysaccharides that affect fibroblast cell adhesion. ACS Appl. Mater. Interfaces.

[135] Zhang M., Deng F., Tang L., Wu H., Ni Y., Chen L., Huang L., Hu X., Lin S., Ding C. (2021). Super-ductile, injectable, fast self-healing collagen-based hydrogels with multi-responsive and accelerated wound-repair properties. Chem. Eng. J..

[136] Che Y., Li D., Liu Y., Yue Z., Zhao J., Ma Q., Zhang Q., Tan Y., Yue Q., Meng F. (2018). Design and fabrication of a triple-responsive chitosan-based hydrogel with excellent mechanical properties for controlled drug delivery. J. Polym. Res..

[137] Wang Z., Si Y., Zhao C., Yu D., Wang W., Sun G. (2019). Flexible and washable poly (ionic liquid) nanofibrous membrane with moisture proof pressure sensing for real-life wearable electronics. ACS Appl. Mater. Interfaces.

[138] Qu X., Zhao Y., Chen Z.A., Wang S., Ren Y., Wang Q., Shao J., Wang W., Dong X. (2021). Thermoresponsive Lignin-Reinforced Poly (Ionic Liquid) Hydrogel Wireless Strain Sensor. Research.

[139] Kuddushi M., Ray D., Aswal V., Hoskins C., Malek N. (2020). Poly (vinyl alcohol) and functionalized ionic liquid-based smart hydrogels for doxorubicin release. ACS Appl. Bio Mater..

[140] Xiang S., Zheng F., Chen S., Lu Q. (2021). Self-healable, recyclable, and ultrastrong adhesive ionogel for multifunctional strain sensor. ACS Appl. Mater. Interfaces.

[141] Kuddushi M., Patel N.K., Rajput S., El Seoud O.A., Mata J.P., Malek N.I. (2020). Temperature-Responsive Low Molecular Weight Ionic Liquid Based Gelator: An Approach to Fabricate an Anti-Cancer Drug-Loaded Hybrid Ionogel. ChemSystemsChem.

[142] Zhang X., Cui C., Chen S., Meng L., Zhao H., Xu F., Yang J. (2022). Adhesive Ionohydrogels Based on Ionic Liquid/Water Binary Solvents with Freezing Tolerance for Flexible Ionotronic Devices. Chem. Mater..

[143] Rahimi M., Shafiei-Irannejad V., Safa K.D., Salehi R. (2018). Multi-branched ionic liquid-chitosan as a smart and biocompatible nano-vehicle for combination chemotherapy with stealth and targeted properties. Carbohydr. Polym..

[144] Correia D.M., Fernandes L.C., Martins P.M., García-Astrain C., Costa C.M., Reguera J., Lanceros-Méndez S. (2020). Ionic liquid—Polymer composites: A new platform for multifunctional applications. Adv. Funct. Mater..

[145] Hu X., Huang J., Zhang W., Li M., Tao C., Li G. (2008). Photonic Ionic Liquids Polymer for Naked-Eye Detection of Anions. Adv. Mater..

[146] Usuba M., Hongo C., Matsumoto T., Nishino T. (2018). On-demand easy peeling of acrylic adhesives containing ionic liquids through a microwave irradiation stimulus. Polym. J..

[147] Li Z., Wang J., Hu R., Lv C., Zheng J. (2019). A highly ionic conductive, healable, and adhesive polysiloxane—Supported ionogel. Macromol. Rapid Commun..

[148] Yu Y., Xie F., Gao X., Zheng L. (2021). Double-network hydrogels with adjustable surface morphology and multifunctional integration for flexible strain sensors. Soft Matter.

[149] Zhang D., Tang Y., Zhang Y., Yang F., Liu Y., Wang X., Yang X., Zheng J. (2020). Highly stretchable, self-adhesive, biocompatible, conductive hydrogels as fully polymeric strain sensors. J. Mater. Chem. A.

[150] Pagano C., Marinozzi M., Baiocchi C., Beccari T., Calarco P., Ceccarini M.R., Chielli M., Orabona C., Orecchini E., Ortenzi R. (2020). Bioadhesive Polymeric Films Based on Red Onion Skins Extract for Wound Treatment: An Innovative and Eco-Friendly Formulation. Molecules.

[151] Zheng K., Gu Q., Zhou D., Zhou M., Zhang L. (2021). Recent progress in surgical adhesives for biomedical applications. Smart Mater. Med..

[152] Xiong Y., Zhang X., Ma X., Wang W., Yan F., Zhao X., Chu X., Xu W., Sun C. (2021). A review of the properties and applications of bioadhesive hydrogels. Polym. Chem..

[153] Brahmbhatt D. (2017). Bioadhesive drug delivery systems: Overview and recent advances. Int. J. Chem. Life Sci..

[154] Jiao Y., Pang X., Liu M., Zhang B., Li L., Zhai G. (2016). Recent progresses in bioadhesive microspheres via transmucosal administration. Colloids Surf. B Biointerfaces.

[155] Deng J., Yuk H., Wu J., Varela C.E., Chen X., Roche E.T., Guo C.F., Zhao X. (2020). Electrical bioadhesive interface for bioelectronics. Nat. Mater..

[156] Wang X., Sun X., Gan D., Soubrier M., Chiang H.-Y., Yan L., Li Y., Li J., Yu S., Xia Y. (2022). Bioadhesive and conductive hydrogel-integrated brain-machine interfaces for conformal and immune-evasive contact with brain tissue. Matter.

[157] Zhao X., Liang Y., Huang Y., He J., Han Y., Guo B. (2020). Physical double—Network hydrogel adhesives with rapid shape adaptability, fast self—Healing, antioxidant and NIR/pH stimulus—Responsiveness for multidrug—Resistant bacterial infection and removable wound dressing. Adv. Funct. Mater..

[158] Liang Y., Li Z., Huang Y., Yu R., Guo B. (2021). Dual-Dynamic-Bond Cross-Linked Antibacterial Adhesive Hydrogel Sealants with On-Demand Removability for Post-Wound-Closure and Infected Wound Healing. ACS Nano.

[159] Liang Y., Zhao X., Hu T., Chen B., Yin Z., Ma P.X., Guo B. (2019). Adhesive Hemostatic Conducting Injectable Composite Hydrogels with Sustained Drug Release and Photothermal Antibacterial Activity to Promote Full—Thickness Skin Regeneration During Wound Healing. Small.

[160] Yan Y.H., Rong L.H., Ge J., Tiu B.D.B., Cao P.F., Advincula R.C. (2019). Mussel—Inspired hydrogel composite with multi—Stimuli responsive behavior. Macromol. Mater. Eng..

[161] Zhu Y., Zeng Q., Zhang Q., Li K., Shi X., Liang F., Han D. (2020). Temperature/near-infrared light-responsive conductive hydrogels for controlled drug release and real-time monitoring. Nanoscale.

[162] Blacklow S.O., Li J., Freedman B.R., Zeidi M., Chen C., Mooney D.J. (2019). Bioinspired mechanically active adhesive dressings to accelerate wound closure. Sci. Adv..

[163] Chang W.C., Tai A.Z., Tsai N.Y., Li Y.C.E. (2021). An Injectable Hybrid Gelatin Methacryloyl (GelMA)/Phenyl Isothiocyanate-Modified Gelatin (Gel-Phe) Bioadhesive for Oral/Dental Hemostasis Applications. Polymers.

[164] Guo Y., Wang Y., Zhao X., Li X., Wang Q., Zhong W., Mequanint K., Zhan R., Xing M., Luo G. (2021). Snake extract–laden hemostatic bioadhesive gel cross-linked by visible light. Sci. Adv..

[165] Zhu Y., Lin L., Chen Y., Song Y., Lu W.P., Guo Y. (2020). A self-healing, robust adhesion, multiple stimuli-response hydrogel for flexible sensors. Soft Matter.

[166] Schnabel B., Scharf M., Schwieger K., Windolf M., van der Pol B., Braunstein V., Appelt A. (2009). Biomechanical comparison of a new staple technique with tension band wiring for transverse patella fractures. Clin. Biomech.

[167] Rathi S., Saka R., Domb A.J., Khan W. (2019). Protein—Based bioadhesives and bioglues. Polym. Adv. Technol..

[168] Rahimnejad M., Zhong W. (2017). Mussel-inspired hydrogel tissue adhesives for wound closure. RSC Adv..

[169] Elliott D.S., Newman K.J.H., Forward D.P., Hahn D.M., Ollivere B., Kojima K., Handley R., Rossiter N., Wixted J., Moran C.G. (2016). A unified theory of bone healing and nonunion: BHN theory. Bone Jt. J..

[170] Shirvan A.R., Bashari A., Hemmatinejad N. (2019). New insight into the fabrication of smart mucoadhesive buccal patches as a novel controlled-drug delivery system. Eur. Polym. J..

[171] Hanafy N.A.N., Leporatti S., El-Kemary M. (2019). Mucoadhesive hydrogel nanoparticles as smart biomedical drug delivery system. Appl. Sci..

[172] Zheng D., Bai B., Zhao H., Xu X., Hu N., Wang H. (2021). Stimuli-responsive Ca-alginate-based photothermal system with enhanced foliar adhesion for controlled pesticide release. Colloids Surf. B Biointerfaces.

[173] Qu M., Jiang X., Zhou X., Wang C., Wu Q., Ren L., Zhu J., Zhu S., Tebon P., Sun W. (2020). Stimuli-Responsive Delivery of Growth Factors for Tissue Engineering. Adv. Healthc. Mater..

[174] Abdollahiyan P., Baradaran B., de la Guardia M., Oroojalian F., Mokhtarzadeh A. (2020). Cutting-edge progress and challenges in stimuli responsive hydrogel microenvironment for success in tissue engineering today. J. Control. Release.

[175] Epstein N. (2014). Hemostasis and other benefits of fibrin sealants/glues in spine surgery beyond cerebrospinal fluid leak repairs. Surg. Neurol. Int..

[176] Wu J., Yuk H., Sarrafian T.L., Guo C.F., Griffiths L.G., Nabzdyk C.S., Zhao X. (2022). An off-the-shelf bioadhesive patch for sutureless repair of gastrointestinal defects. Sci. Transl. Med..

[177] Kiratli H., Tarlan B. (2013). Subconjunctival hemorrhage: Risk factors and potential indicators. Clin. Ophthalmol..

[178] Lee G.-H., Moon H., Kim H., Lee G.H., Kwon W., Yoo S., Myung D., Yun S.H., Bao Z., Hahn S.K. (2020). Multifunctional materials for implantable and wearable photonic healthcare devices. Nat. Rev. Mater..

[179] Chen X., Yuk H., Wu J., Nabzdyk C.S., Zhao X. (2020). Instant tough bioadhesive with triggerable benign detachment. Proc. Natl. Acad. Sci. USA.

[180] Ionov L. (2014). Hydrogel-based actuators: Possibilities and limitations. Mater. Today.

[181] Wang M., Zhai Y., Ye H., Lv Q., Sun B., Luo C., Jiang Q., Zhang H., Xu Y., Jing Y. (2019). High Co-loading Capacity and Stimuli-Responsive Release Based on Cascade Reaction of Self-Destructive Polymer for Improved Chemo-Photodynamic Therapy. ACS Nano.

[182] Zheng F., Xiong W., Sun S., Zhang P., Zhu J.J. (2019). Recent advances in drug release monitoring. Nanophotonics.

[183] Naseem F., Shah S.U., Rashid S.A., Farid A., Almehmadi M., Alghamdi S. (2022). Metronidazole Based Floating Bioadhesive Drug Delivery System for Potential Eradication of H. pylori: Preparation and In Vitro Characterization. Polymers.

[184] Céspedes-Valenzuela D.N., Sánchez-Rentería S., Cifuentes J., Gantiva-Diaz M., Serna J.A., Reyes L.H., Ostos C., la Portilla C.C.-D., Muñoz-Camargo C., Cruz J.C. (2021). Preparation and Characterization of an Injectable and Photo-Responsive Chitosan Methacrylate/Graphene Oxide Hydrogel: Potential Applications in Bone Tissue Adhesion and Repair. Polymers.

[185] Wong Y.L., Pandey M., Choudhury H., Lim W.M., Bhattamisra S.K., Gorain B. (2021). Development of In-Situ Spray for Local Delivery of Antibacterial Drug for Hidradenitis Suppurativa: Investigation of Alternative Formulation. Polymers.

[186] Spiridon I., Andrei I.-M., Anghel N., Dinu M., Ciubotaru B.-I. (2021). Development and Characterization of Novel Cellulose Composites Obtained in 1-Ethyl-3-methylimidazolium Chloride Used as Drug Delivery Systems. Polymers.

[187] Ageitos J., Robla S., Valverde-Fraga L., Garcia-Fuentes M., Csaba N. (2021). Purification of Hollow Sporopollenin Microcapsules from Sunflower and Chamomile Pollen Grains. Polymers.

[188] Anghel N., Dinu V., Verestiuc L., Spiridon I. (2021). Transcutaneous Drug Delivery Systems Based on Collagen/Polyurethane Composites Reinforced with Cellulose. Polymers.

[189] Antov P., Krišt’ák L., Réh R., Savov V., Papadopoulos A.N. (2021). Eco-Friendly Fiberboard Panels from Recycled Fibers Bonded with Calcium Lignosulfonate. Polymers.

[190] Nifant’ev I., Shlyakhtin A., Komarov P., Tavtorkin A., Kananykhina E., Elchaninov A., Vishnyakova P., Fatkhudinov P., Ivchenko P. (2020). In Vitro and In Vivo Studies of Biodegradability and Biocompatibility of Poly (εCL)-b-Poly (EtOEP)-Based Films. Polymers.

[191] Saha N., Saha N., Sáha T., Öner E.T., Brodnjak U., Redl H., Von Byern J., Sáha P. (2020). Polymer Based Bioadhesive Biomaterials for Medical Application—A Perspective of Redefining Healthcare System Management. Polymers.

[192] Rusu L.-C., Ardelean L.C., Jitariu A.-A., Miu C.A., Streian C.G. (2020). An Insight into the Structural Diversity and Clinical Applicability of Polyurethanes in Biomedicine. Polymers.

[193] Spiridon I., Anghel N., Dinu M.V., Vlad S., Bele A., Ciubotaru B.-I., Verestiuc L., Pamfil D. (2020). Development and Performance of Bioactive Compounds-Loaded Cellulose/Collagen/Polyurethane Materials. Polymers.

[194] Wu Y., Rashidpour A., Almajano M.P., Metón I. (2020). Chitosan-Based Drug Delivery System: Applications in Fish Biotechnology. Polymers.

[195] Krabicová I., Appleton S.L., Tannous M., Hoti G., Caldera F., Pedrazzo A.R., Cecone C., Cavalli R., Trotta F. (2020). History of Cyclodextrin Nanosponges. Polymers.

[196] Wei S.-M., Pei M.-Y., Pan W.-L., Thissen H., Tsai S.-W. (2020). Gelatin Hydrogels Reinforced by Absorbable Nanoparticles and Fibrils Cured In Situ by Visible Light for Tissue Adhesive Applications. Polymers.

[197] Pahlevanzadeh F., Emadi R., Valiani A., Kharaziha M., Poursamar S.A., Bakhsheshi-Rad H.R., Ismail A.F., RamaKrishna S., Berto F. (2020). Three-dimensional printing constructs based on the chitosan for tissue regeneration: State of the art, developing directions and prospect trends. Materials.

[198] Elahpour N., Pahlevanzadeh F., Kharaziha M., Bakhsheshi-Rad H.R., Ramakrishna S., Berto F. (2021). 3D printed microneedles for transdermal drug delivery: A brief review of two decades. Int. J. Pharm..

[199] Pahlevanzadeh F., Mokhtari H., Bakhsheshi-Rad H.R., Emadi R., Kharaziha M., Valiani A., Poursamar S.A., Ismail A.F., RamaKrishna S., Berto F. (2020). Recent trends in three-dimensional bioinks based on alginate for biomedical applications. Materials.

[200] Pahlevanzadeh F., Bakhsheshi-Rad H.R., Kharaziha M., Kasiri-Asgarani M., Omidi M., Razzaghi M., Ismail A.F., Sharif S., RamaKrishna S., Berto F. (2021). CNT and rGO reinforced PMMA based bone cement for fixation of load bearing implants: Mechanical property and biological response. J. Mech. Behav. Biomed. Mater..

[201] Pahlevanzadeh F., Bakhsheshi-Rad H.R., Brabazon D., Kharaziha M., Ismail A.F., Sharif S., Razzaghi M., Berto F. (2021). Additive Manufacturing of Polymer Matrix Composites.

[202] Mokhtari H., Tavakoli S., Safarpour F., Kharaziha M., Bakhsheshi-Rad H.R., Ramakrishna S., Berto F. (2021). Recent advances in chemically-modified and hybrid carrageenan-based platforms for drug delivery, wound healing, and tissue engineering. Polymers.

[203] Rajabi N., Rezaei A., Kharaziha M., Bakhsheshi-Rad H.R., Luo H., RamaKrishna S., Berto F. (2021). Recent advances on bioprinted gelatin methacrylate-based hydrogels for tissue repair. Tissue Eng. Part A.

[204] Bai Y., Liu X., Shi S.Q., Li J. (2020). A Tough and Mildew-Proof Soybean-Based Adhesive Inspired by Mussel and Algae. Polymers.

